# Cross-domain transfer of trehalose biosynthesis genes contributes to adaptation in high-altitude environments

**DOI:** 10.1093/nsr/nwag117

**Published:** 2026-02-25

**Authors:** Yaohai Wang, Jiahao Ni, Jiao Pan, Ruobing Feng, Weiyi Li, Xuetao Zhang, Chao Gao, Lijian Liao, Zhirong Zhang, Hongwei Yue, Kexin Zhang, Lin Zhang, Chunhui Feng, Dongji Yao, Yumin Han, Xunrong Li, Xuan Zhou, Ziguang Deng, Jia Zhang, Pin Zhou, Gongchao Jing, Yu Zhang, Lingyun Chen, Xuming Pan, Xiangrui Chen, Yang Bai, Ying Yan, Jie Huang, Zhiqiang Ye, Xiaopeng Shen, Miao Tian, Rebecca A Zufall, Pingyuan Wang, Michael Lynch, Hongan Long

**Affiliations:** Key Laboratory of Evolution and Marine Biodiversity (Ministry of Education), Institute of Evolution and Marine Biodiversity, Ocean University of China, Qingdao 266003, China; Laboratory for Marine Biology and Biotechnology, Qingdao Marine Science and Technology Center, Qingdao 266237, China; Key Laboratory of Evolution and Marine Biodiversity (Ministry of Education), Institute of Evolution and Marine Biodiversity, Ocean University of China, Qingdao 266003, China; Key Laboratory of Evolution and Marine Biodiversity (Ministry of Education), Institute of Evolution and Marine Biodiversity, Ocean University of China, Qingdao 266003, China; Key Laboratory of Evolution and Marine Biodiversity (Ministry of Education), Institute of Evolution and Marine Biodiversity, Ocean University of China, Qingdao 266003, China; Department of Genetics, Stanford University School of Medicine, Stanford, CA 94305, USA; Key Laboratory of Marine Drugs, the Ministry of Education of China, School of Medicine and Pharmacy, Ocean University of China, Qingdao 266003, China; Key Laboratory of Evolution and Marine Biodiversity (Ministry of Education), Institute of Evolution and Marine Biodiversity, Ocean University of China, Qingdao 266003, China; Key Laboratory of Evolution and Marine Biodiversity (Ministry of Education), Institute of Evolution and Marine Biodiversity, Ocean University of China, Qingdao 266003, China; Key Laboratory of Evolution and Marine Biodiversity (Ministry of Education), Institute of Evolution and Marine Biodiversity, Ocean University of China, Qingdao 266003, China; Key Laboratory of Evolution and Marine Biodiversity (Ministry of Education), Institute of Evolution and Marine Biodiversity, Ocean University of China, Qingdao 266003, China; Key Laboratory of Evolution and Marine Biodiversity (Ministry of Education), Institute of Evolution and Marine Biodiversity, Ocean University of China, Qingdao 266003, China; Key Laboratory of Evolution and Marine Biodiversity (Ministry of Education), Institute of Evolution and Marine Biodiversity, Ocean University of China, Qingdao 266003, China; Key Laboratory of Evolution and Marine Biodiversity (Ministry of Education), Institute of Evolution and Marine Biodiversity, Ocean University of China, Qingdao 266003, China; Key Laboratory of Evolution and Marine Biodiversity (Ministry of Education), Institute of Evolution and Marine Biodiversity, Ocean University of China, Qingdao 266003, China; Key Laboratory of Evolution and Marine Biodiversity (Ministry of Education), Institute of Evolution and Marine Biodiversity, Ocean University of China, Qingdao 266003, China; Qingdao Institute of Bioenergy and Bioprocess Technology, Chinese Academy of Sciences, Qingdao 266101, China; Qingdao Institute of Bioenergy and Bioprocess Technology, Chinese Academy of Sciences, Qingdao 266101, China; Key Laboratory of Evolution and Marine Biodiversity (Ministry of Education), Institute of Evolution and Marine Biodiversity, Ocean University of China, Qingdao 266003, China; Qingdao Institute of Bioenergy and Bioprocess Technology, Chinese Academy of Sciences, Qingdao 266101, China; Qingdao Single-Cell Biotech. Co., Ltd., Qingdao 266100, China; Qingdao Institute of Bioenergy and Bioprocess Technology, Chinese Academy of Sciences, Qingdao 266101, China; Key Laboratory of Evolution and Marine Biodiversity (Ministry of Education), Institute of Evolution and Marine Biodiversity, Ocean University of China, Qingdao 266003, China; College of Life Science, Northwest Normal University, Lanzhou 730070, China; Laboratory of Protozoology, Harbin Normal University, Harbin 150025, China; School of Marine Science, Ningbo University, Ningbo 315211, China; Ministry of Education Key Laboratory of Ecology and Resource Use of the Mongolia Plateau, Inner Mongolia University, Hohhot 010021, China; Key Laboratory of Evolution and Marine Biodiversity (Ministry of Education), Institute of Evolution and Marine Biodiversity, Ocean University of China, Qingdao 266003, China; State Key Laboratory of Lake and Watershed Science for Water Security, Institute of Hydrobiology, Chinese Academy of Sciences, Wuhan 430072, China; School of Life Sciences, Central China Normal University, Wuhan 430079, China; College of Life Sciences, Anhui Normal University, Wuhu 241000, China; Key Laboratory of Evolution and Marine Biodiversity (Ministry of Education), Institute of Evolution and Marine Biodiversity, Ocean University of China, Qingdao 266003, China; Department of Biology and Biochemistry, University of Houston, Houston TX 77204, USA; Key Laboratory of Evolution and Marine Biodiversity (Ministry of Education), Institute of Evolution and Marine Biodiversity, Ocean University of China, Qingdao 266003, China; Biodesign Center for Mechanisms of Evolution, Arizona State University, Tempe AZ 85287, USA; Key Laboratory of Evolution and Marine Biodiversity (Ministry of Education), Institute of Evolution and Marine Biodiversity, Ocean University of China, Qingdao 266003, China; Laboratory for Marine Biology and Biotechnology, Qingdao Marine Science and Technology Center, Qingdao 266237, China

**Keywords:** ciliate, evolutionary genomics, extremophile, high-altitude adaptation

## Abstract

High altitudes pose extreme survival challenges for organisms, yet the origins and molecular strategies underlying their resilience remain poorly understood. Here, we report the molecular and evolutionary mechanisms underlying stress resilience in *Apourosomoida* sp. LHA081A01, a ciliate isolated from a high-altitude Tibetan salt lake that endures high salinity, low temperature, and hypoxia. We identified TreT glycosyltransferases, acquired through horizontal gene transfer from an anaerobic and halophilic Desulfobacteraceae bacterium, to be involved in the synthesis of α,α-trehalose—a universal protein stabilizer absent in most other ciliates but essential for counteracting multiple environmental stressors. Additional strategies include β-carotene accumulation to mitigate oxidative stress from hypoxia, along with numerous others common to many eukaryotes. Extensive gene family expansions and rapid divergence of stress‑responsive genes underscore their evolutionary significance and critical role in surviving harsh habitats. Intolerance to low salinity may render this ciliate, and other protists, vulnerable to climate‑driven salinity declines in Tibetan salt lakes. Together, these extraordinary features—shaped by horizontal gene transfer, natural selection, and regulatory plasticity—position high-altitude microbial eukaryotes as powerful extremophile models for uncovering the molecular mechanisms of stress resilience and adaptive evolution across life.

## INTRODUCTION

Many stress-resilience mechanisms have been found in extremophiles in high altitudes, enabling survival in some of the most challenging environments on Earth. For example, the high proportion of long-chain polyunsaturated fatty acids in the lipid bilayer of cell membranes helps maintain membrane fluidity in high‑mountain plants at low temperatures [1]. Highland native humans have enhanced lung volume/function and improved oxygen transport/utilization to cope with low oxygen levels [2]. Yeasts isolated from high‑altitude volcanic areas in the Atacama Desert contain cellular carotenoids and melanin that absorb different UV wavelengths and remove free radicals generated by UV radiation [3]. Additionally, Archaea from Andean salt lakes store high concentrations of potassium ions and produce halo‑compatible proteins to survive extreme salinity, among other adaptations [4,5]. Understanding

how organisms survive such extreme conditions—particularly those capable of surviving and thriving in multiple extreme environmental conditions simultaneously (polyextremophiles)—can provide valuable insights into the limits of life and potential strategies for coping with additional stresses imposed by global climate change.

The Tibetan Plateau, also known as the Qinghai-Tibet Plateau (QTP) or the ‘Roof of the World’, is the highest and largest plateau on Earth, rising over 4500 meters above sea level [6]. Formed by the immense collision of the Indian and Eurasian tectonic plates ∼50 million years ago, it continues its slow ascent, shaping one of the planet’s most extreme and enigmatic landscapes [7]. Scattered across this vast expanse are thousands of lakes, including numerous salt lakes such as Lake Qinghai, which were formed through intense evaporation and mineral accumulation [8]. These otherworldly environments—characterized by high salinity and heavy metal ions, low oxygen, intense UV radiation, and extreme temperature fluctuations—serve as crucibles of evolutionary innovation, probably fostering the emergence of uniquely adapted polyextremophiles capable of surviving multiple stressors, different from those previously reported in other habitats [9,10]. Yet, hidden within these saline depths, the vast and diverse world of microbial eukaryotes, particularly protozoa, remains largely unexplored. This neglect stems in part from technical challenges, including limited genetic tools, scarce high-quality genomic resources, and difficulties in sampling and culturing, as well as the research scope of the small protozoological research community. As a result, the survival strategies and evolution of protozoa in Tibetan salt lakes remain a mystery, despite their high level of diversity, ecological significance, and sensitivity to environmental changes [11,12].

Ciliates are protozoa characterized by their covering of cilia and nuclear dimorphism [13]: a transcriptionally inactive micronucleus, which functions exclusively in sexual reproduction, and a macronucleus, responsible for gene expression during vegetative growth—both sequestered within the same cytoplasm. They are widely distributed in most habitats and occupy pivotal niches in microbial food loops and biogeochemical cycles. Despite this, little in-depth research has been reported on free-living ciliates successfully inhabiting extreme environments, some exceptions including anaerobic ciliates in soil and sediments of various water bodies [14,15], and high-salinity-tolerant species with tiny genome size [16]. For ciliates living in even harsher habitats particularly in high-altitude salt lakes, which pose great challenges from high salinity, low oxygen, and temperature fluctuations, their survival strategies remain unexplored.

**Figure 1.**
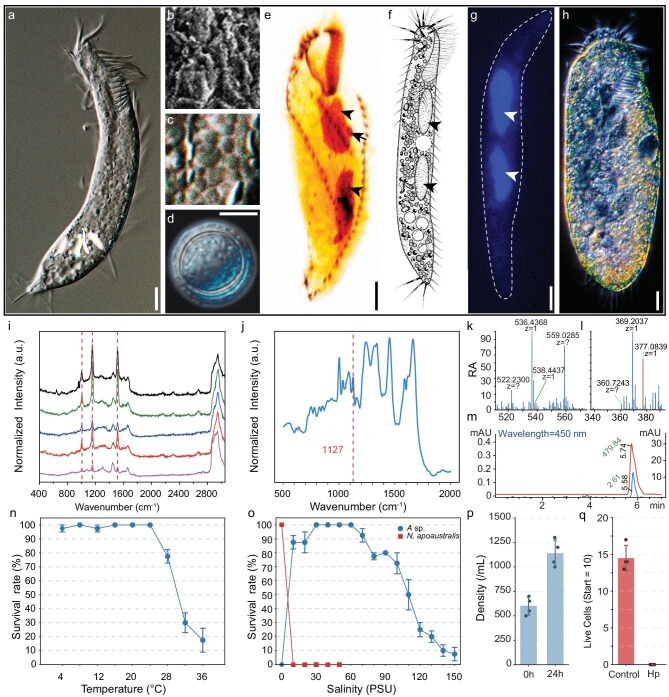
Morphological features, physiological responses, and survival assays of *Apourosomoida* sp. (a–g) Morphological features. (a, f) Ventral view. (b) SEM image of wrinkled cortex. (c) Magnified view of (a), showing the scaly surface of a living cell. (d) Resting cyst. (e–g) Macronuclei (arrowheads) and micronucleus (arrow). (h) Ventral view of *Notohymena apoaustralis*. (i) Normalized Raman spectra of five randomly chosen cells (red dashed lines show β-carotene peaks). (j) Normalized Raman spectrum showing the trehalose peak (red dashed lines, the curve is based on mean values of all measured cells). (k and l) HRESIMS of sample solution 1 (positive ion mode, k) and sample solution 2 (negative ion mode, l). The x-axis represents the mass-to-charge ratio (m/z), and the y-axis indicates relative abundance (RA). In each spectrum, the red peak marks the characteristic m/z value corresponding to β-carotene (m/z 538.4437, k) and the [M + Cl]⁻ adduct ion of trehalose (m/z 377.0839, l), respectively. (m) Chromatograms were recorded at a detection wavelength of 450 nm, with absorbance (y-axis, mAU: milliabsorbance units) plotted against retention time (x-axis, min). The red curve represents the β-carotene commercial product standard, with a major peak at 5.78 min (peak areas indicated in green text, units: mAU·s). The blue curve corresponds to the *Apourosomoida* sp. extract, showing multiple peaks with one aligning to the retention time of the β-carotene standard. Data were processed with baseline subtraction, and peak area units (mAU·s) are displayed directly on the chromatogram in green text. (n) Survival assay across a temperature gradient (4–36°C, 1 h). (o) Differential salinity tolerance between *Apourosomoida* sp. (blue circles) and *N. apoaustralis* (red squares). Survival rates were measured after 1 h exposure to a salinity gradient (0–150 PSU). Data points represent mean survival counts (10 starting cells); error bars indicate SEM. (p) Cell density of *Apourosomoida* sp. measured before hypoxia treatment and after 24 h of hypoxia treatment (0.5 mg/L, 24 h). (q) Survival of *N. apoaustralis* (starting with 10 cells) incubated under normoxic (Control) and hypoxic (Hp) conditions for 24 h. No survivors were detected in the hypoxic group. Scale bars: 10 μm (a, d, e, g, and h).

During a recent biodiversity survey of ciliates in Tibetan salt lakes, we isolated a free-living spirotrich ciliate *Apourosomoida* sp. LHA081A01, from the salt lake Yibug Caka and successfully established a stable cell culture (Fig. 1a). *Apourosomoida* was originally reported by Foissner *et al.* [17] and known to be especially tolerant to high salinity; however, it has not been previously studied at the genetic or genomic level. Members of the class Spirotrichea display a distinct genomic architecture characterized by the extensive fragmentation of the macronucleus (MAC). These MAC genomes consist of ‘gene-sized’ chromosomes—often referred to as nanochromosomes—which typically range from 1 kbp to 3.5 kbp in length and predominantly encode 1–2 genes [18–20]. Yet, despite the immense biodiversity of Spirotrichea, genomic resources for this group remain scarce.

The isolation of this species provides us with a unique opportunity to explore the stress-resilience mechanisms of polyextremophilic protozoa in high-altitude salt lakes while addressing these genomic knowledge gaps. We conducted a comprehensive study of the species, including its morphology, biochemical composition, macronuclear genomics, comparative genomics, physiological responses and gene expression/functional validation under various environmental conditions. Additionally, we explored the origin and evolution of key genes associated with its polyextremophilic capabilities. This study not only advances our understanding of polyextremophilic mechanisms, but also offers valuable insights into the evolution of neglected microbial eukaryotes in extreme environments.

## RESULTS

### Polyextremophilic capabilities of *Apourosomoida* sp. LHA081A01

Halophilic species of *Apourosomoida* were previously reported in saline habitats of Portugal, Australia, and Namibia [21]. The Yibug Caka isolate represents the first documentation of this genus in Asia and, to the best of our knowledge, its stable cell line is also the only living one in the world. The cells are slender and transparent, with a mean *in vivo* size of 116 × 23 μm, two macronuclei, one micronucleus, and scaly and wrinkled cell cortex (Fig. 1a–g and Table S1). Raman spectroscopy analyses revealed the presence of β-carotene and trehalose in the cells (Fig. 1i and j), indicating their intracellular accumulation. These molecules were further confirmed by high-resolution electrospray ionization mass spectrometry (HRESIMS) (Fig. 1k and l), and HPLC (Fig. 1m). Both molecules are known powerful antioxidants, capable of removing free radicals generated under stresses such as high salinity, extreme temperatures, and low oxygen conditions [22–24]. Trehalose, in particular, is well-known as an important protein stabilizer and widely regarded as a universal anti-stress molecule that protects cells and biomolecules from damage caused by various environmental stressors [25–28]. To our knowledge, among ciliates, trehalose synthesis has only been documented in the soil species* Colpoda cucullus* [29]. The evolutionary origin of the trehalose synthesis genes in ciliates remains unknown.

Under short-term temperature fluctuations, cell survival remains stable even at temperatures as low as 4°C but begins to decline at 24°C and above (Fig. 1n and Table S2). This is possibly shaped by the predominantly frigid state of the lake throughout the year (with an average annual air temperature of −6 to −4°C) [30]. Similarly, cells show extraordinary salinity tolerance, surviving within a range of 10–150 PSU (Practical Salinity Unit), with an optimal salinity between 30 and 60 PSU (Fig. 1o and Table S2). The wide tolerance range is consistent with the high salinity of the lake (∼50 PSU) and the frequent freshwater influx from precipitation and melting ice. However, cells rupture in freshwater and perish within 1 h of exposure. Even at low salinity (5–10 PSU), cells transform into resting cysts, a state typically associated with starvation (Fig. 1d).

Even more strikingly, population density continued to increase even at a dissolved O_2_ (DO) concentration of 0.50 mg/L—about 10 times lower than the original sample concentration (5.36 mg/L) (Fig. 1p and Table S2). In contrast, its freshwater relative *N. apoaustralis*, isolated from a low-altitude environment (29 m; DO 10.92 mg/L), showed much lower tolerance to environmental stresses. It failed to survive under hypoxic conditions (DO <0.5 mg/L; Fig. 1q and Table S2), and likewise did not survive at salinity levels exceeding 10 PSU (Fig. 1o and Table S2). For a broader comparison of hypoxia resilience across ciliates, aerobic ciliates typically thrive at DO ≥6.5 mg/L and show significant growth inhibition when DO drops below ∼1.25 mg/L [31], whereas anaerobic ciliates thrive in strictly anoxic environments but fail to grow under aerobic conditions [32,33]. These findings demonstrate that *Apourosomoida* sp. LHA081A01 is a true polyextremophile, capable of withstanding extreme levels of temperature, oxygen, and salinity.

### Distinctive genomic features for survival in the high-altitude salt lake

**Figure 2.**
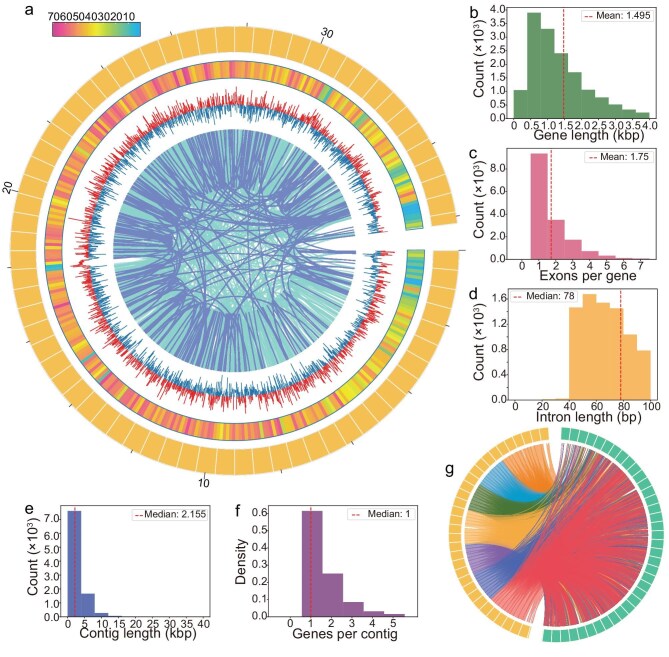
*Apourosomoida* sp. genome assembly and comparative analysis. (a) Circos plot of genomic features: outer to inner layers show genome, gene density (per 0.1 Mbp), GC skew calculated in 20 kbp sliding windows (5 kbp step size), and homologous gene pairs (dark blue regions indicate significant DEGs identified under stress conditions). (b) Length distribution of protein-coding genes. (c) Distribution of exon numbers per gene. (d) Length distribution of introns (<100 bp). (e) Length distribution of assembled contigs. (f) Distribution of gene counts per contig. (g) Homologous gene pairs between *Apourosomoida* sp. (yellow) and *Notohymena apoaustralis* (green), colors of collinearity lines are only for avoiding visual clutter.

A high-quality genome is essential for revealing polyextremophilic mechanisms at the molecular level. We thus *de novo* assembled and annotated the macronuclear genome of *Apourosomoida* sp., with the haploid genome size of 36.17 Mbp and a GC content of 38.32% (one of the highest GC contents among ciliates—organisms generally featured by low GC content—second only to the halotolerant *Fabrea salina* [16]; Table S3), N50 of 4694 bp, 15905 protein-coding genes (mean gene size 1495 bp) and 1473 non-coding RNAs, yielding a gene density of ∼480 per Mbp (Fig. 2a and b, Table 1, and Table S3). Genes average 1.75 exons (median length: 240 bp), ∼59% of the genes do not contain any intron and the median intron size is 78 bp (Fig. 2c and d, and Tables S3 and S4). Among the 10474 macronuclear contigs (median length: 2155 bp; Fig. 2e), 61.53% contain only one gene, 25.12% carry two genes (Fig. 2f), 52.55% have telomeric repeats (C_4_A_4_, T_4_G_4_) at both ends, while 32.13% have telomeric repeats at only one end (Table 1). The assembly is characterized by an extremely small N50, with large number of contigs bearing telomeres at both ends—hallmarks of the highly fragmented macronuclear genomes typical of spirotrich ciliates (Table 1), such as the model species *Oxytricha trifalla*x (N50 = 3.74 kbp), *Stylonychia lemnae* (3.29 kbp), and *Euplotes vannus* (2.69 kbp) (Table S3) [18,20,34,35].

**Table 1.** The macronuclear genomic features of *Apourosomoida* sp. LHA081A01 and *Notohymena apoaustralis* SC0818.

**Species**	***Apourosomoida* sp.**	***Notohymena apoaustralis***
Genome size/bp	36181345	41734863
GC content	38.32%	35.61%
Contigs	10475	8683
N50	4694	5374
L50	1883	1649
Contigs with both telomeres	5504 (52.54%)	1856 (21.38%)
Contigs with one telomere	3365 (32.12%)	4306 (49.59%)
BUSCO	86%	80%
Number of contigs with 18S rRNA genes	1	1
Protein-coding genes	15905	18977
Non-coding RNA	1475	2186
rRNA number	6	11
tRNA number	84	18
Nanopore depth	373×	41×

Numerous stress-resilience genes are annotated, such as *PYS, PDS*, and *CrtISO* involved in β-carotenoid biosynthesis—which are absent in most other ciliates, as well as TreT glycosyltransferases in α,α-trehalose reversible synthesis, consistent with our Raman spectroscopy, HRESIMS, and HPLC results, which further confirmed the presence of trehalose and β-carotene in *Apourosomoida* sp. (Fig. 1i–m and Table S5). The P-loop containing nucleoside triphosphate hydrolase, the protein kinase–like domain superfamily, and the zinc finger—RING/FYVE/PHD-type are the three largest gene families in the genome (Table S6). They are known to be involved in responses to osmotic, oxidative, and thermal stresses [36–38]. We also identified additional genes, including a cryptochrome DASH homolog (evm.model.tig3252.1) that senses near-UV/blue light for photoprotection; two key antioxidant enzymes: glutathione peroxidase (evm.model.tig2207.1) for peroxide detoxification and superoxide dismutase (evm.model.tig4611.1) for ROS scavenging; zinc finger A20/AN1 domain proteins, which regulate heat-shock proteins; a cold-induced DEAD-box RNA helicase 38 (evm.model.tig6647.1) that stabilizes transcripts; and a CRE-RAB7 protein (evm.model.tig502.3), which regulates vesicular trafficking to maintain ion homeostasis upon salinity stress (Table S7).

**Figure 3.**
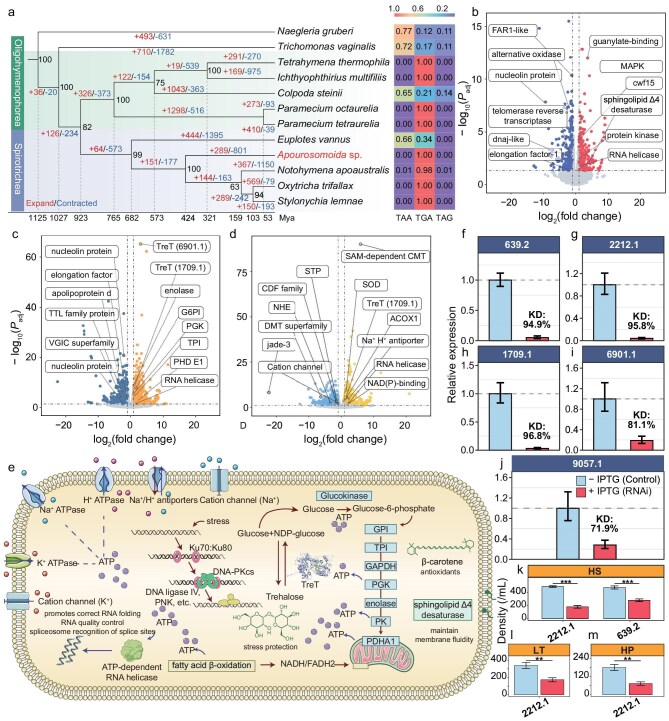
Stress-resilience mechanisms and evolutionary patterns of *Apourosomoida* sp. (a) Gene-family dynamics across 12 ciliate species. Bootstrap support values are displayed at the nodes. The numbers above branches indicate gene family expansion (red, left) and contraction (blue, right) events. The timeline at the bottom represents divergence time (million years ago, Mya). *Tetrahymena thermophila* vs *Paramecium tetraurelia; Ichthyophthirius multifiliis* vs *Tetrahymena thermophila* were used as calibration points in Timetree estimation, and stop codon distributions (right). (b–d) Volcano plots comparing DEGs under treatments: (b) low temperature (4°C) vs control (16°C); (c) hypoxia (0.5 mg/L) vs control (6.87 mg/L); (d) high salinity (80 PSU) vs control (50 PSU). (e) Pathways associated with stress resilience, from genome annotations or differential gene expression analyses: up-regulated glycolysis upon hypoxia, ion transport genes under high salinity, low-temperature expression of sphingolipid Δ4-desaturase, HGT-derived trehalose synthase (TreT), β-carotene biosynthesis, and NHEJ repair. (f–j) RT-qPCR verification of RNAi knockdown efficiency for five candidate genes. Relative expression levels in IPTG-induced groups (+IPTG) are compared to non-induced controls (−IPTG, set to 1). The percentage values on the bars indicate the knockdown efficiency (KD). (k–m) Cell density of *Apourosomoida* sp. following RNAi-mediated knockdown of two genes—protein kinase (evm.model.tig2212.1), and an unannotated gene (evm.model.tig639.2)—under three stressing conditions: low temperature (LT; 4°C), high salinity (HS; 80 PSU), and hypoxia (HP; 0.5 mg/L) for 24 h. Bar colors follow the same scheme as in f–j (blue: −IPTG; red: +IPTG). Statistical significance was calculated using a two-sample *t*-test (^∗∗∗^*P* < 0.001, ^∗∗^*P* < 0.01).

In addition, similar to model ciliates like *Oxytricha trifallax, Tetrahymena thermophila*, and *Paramecium tetraurelia*, stop codon reassignment also occurs, with TGA as the only stop codon, while TAA and TAG are reassigned to code glutamine (Gln; Fig. 3a, Fig. S1a, and Table S8). Further analysis of the relative synonymous codon usage (RSCU) in *Apourosomoida* sp. revealed that the AGG codon (Arg) displayed a distinct preference in *Apourosomoida* sp., a feature not observed in freshwater Spirotrichea species such as *Strombidium lemnae* and *O. trifallax*, but present in salt-tolerant species like *Euplotes octocarinatus* and *Uroleptopsis citrina* [39]. In addition to nuclear genomic features, the mitochondrial genome is also one of the largest among ciliates (83928 bp), containing 64 protein-coding genes, 20 tRNAs, and 2 rRNA genes (Fig. S1b and Table S9).

To find out whether the genomic features were possibly shaped by extreme habitats, we also *de novo* assembled the macronuclear genome of *Notohymena apoaustralis* SC0818, a ciliate isolated from a freshwater pond at an altitude of 29 meters—the closest phylogenetic relative available for comparison (Figs 1h and 3a). The genome of *N. apoaustralis* (41.73 Mbp, GC content 35.61%) contains more protein-coding genes (18977) than *Apourosomoida* sp. (15905), and displays a lower GC content across the genome, exons, and introns (Table 1, and Tables S3 and S4). A total of 7186 homologous gene pairs were identified between the two species, providing a basis for comparative genomic analyses (Fig. 2g). It is worth noting that the β-carotene and trehalose biosynthesis pathways are absent from the *N. apoaustralis* genome, suggesting that these pathways may not be essential in non-extreme environments (Table S5).

### Stress-resilience genes in *Apourosomoida* sp. facilitate responses to extreme environmental challenges through differential expression

To investigate how stress-resilience genes contribute to survival under cold, hypoxia, or hypersaline conditions, we evaluated the transcriptional profiles in response to stress levels spanning nearly the full range of the salt lake fluctuations. At low temperature (4°C), compared with the optimal temperature 16°C, 750 differentially expressed genes (DEGs) were identified (369 down-/381 up-regulated; Fig. 3b, and Tables S10 and S11). Down-regulated DEGs are mainly associated with primary metabolic processes, including lipid metabolism and glycolysis, macromolecule synthesis (e.g. ribosome biogenesis and translational elongation), and catalytic enzymes such as acyltransferases (Fig. S2a and Table S12). By contrast, up-regulated genes are especially enriched in pathways related to environmental sensing, cell cycle checkpoint reinforcement, and post-translational modifications (Fig. S2b and Table S12), potentially reflecting a multi-layered regulatory response to mitigate cold stress. The MAPK pathway, a major and conserved signaling pathway regulating processes stimulated by extracellular factors, is also activated (Fig. S3a), wherein the MAPK cascade, including extracellular regulated protein kinases (ERKs), might transiently elevate intracellular calcium levels, subsequently activating calcium-binding proteins and calcium-dependent protein kinases to maintain cellular activity at cold temperatures. Additionally, the up-regulation of sphingolipid Δ4-desaturase (Fig. S3a), a plasma membrane protein, may contribute to the increase of unsaturated fatty acids, which is consistent with a mechanism that helps maintain cell membrane fluidity at low temperatures [40].

Under hypoxic conditions (0.5 mg/L), deprivation of oxygen, the terminal electron acceptor impairs mitochondrial electron transport chain efficiency. Transcriptomic profiling revealed 1937 DEGs (945 down-/992 up-regulated) (Fig. 3c, and Tables S10 and S13). Down-regulated genes were significantly enriched in biological processes related to translation, such as ribosomal biogenesis, rRNA processing, and tRNA methylation. This may suggest a strategic attenuation of the translational machinery under hypoxic stress to conserve energy. Specifically, the suppression of ribosomal large subunit assembly and tRNA processing suggests a reduction in protein synthesis and translational activities, which would help alleviate the energy burden (Fig. S2c). Meanwhile, the up-regulated genes displayed significant activation of the glycolysis pathway, suggesting a possible metabolic shift toward anaerobic glycolysis under oxygen-limited conditions (Figs S2d and S3c, and Table S12). The up-regulation of a glycosyltransferase (TreT; evm.model.tig1709.1) may enhance cellular response to hypoxia through increasing the synthesis of trehalose, which stabilizes proteins, suppresses ROS-mediated DNA damage, and synergistically balances carbon flux with hypoxia-induced glycolytic enzymes (GPI, PGK) to maintain ATP production while accumulating osmoprotectants. These pathways integrate metabolic remodeling and stress protection to collectively maintain cellular homeostasis under low-oxygen conditions.

Under hypersaline conditions (80 PSU), which potentially impose survival challenges through ion toxicity, osmotic imbalance, and membrane rigidity—transcriptomic analysis revealed 1606 DEGs (756 down-/850 up-regulated) (Fig. 3d, and Tables S10 and S14). Maintaining a high Na⁺ gradient is fundamental for the survival of halophilic organisms. In *Apourosomoida* sp., a plasma membrane Na⁺/H⁺ antiporter (Fig. S3e) was identified and demonstrated significant up-regulation under high-salinity conditions, suggesting coordinated regulation of ion-transport systems as a critical survival strategy under hypersaline stress. This parallels findings in the halophilic alga *Dunaliella salina* elevating the expression of Na⁺/H⁺ antiporters in response to rising salinity levels [41]. In contrast, genes significantly down-regulated under hypersaline treatment were enriched in the monoatomic cation transmembrane transport, indicating a suppression of certain ion-transport activities. This may reflect a strategic reduction in both K⁺ efflux and Na⁺ influx to help maintain a high intracellular K⁺/Na⁺ ratio—an essential component of salt tolerance in many halophilic organisms [42]. Also, the suppression of genes associated with DNA replication/repair (including components of the MCM complex), chromatin assembly, and the cell cycle implies that cells might enter transient cell-cycle arrest and structural reorganization to conserve energy and prevent DNA damage. The enhanced activity of the fatty acid β-oxidation pathway might efficiently break down fatty acids to generate acetyl-CoA, which enters the TCA cycle to produce ATP (Fig. S4a and Table S12). Additionally, the resulting NADH/FADH2 may help sustain the electron transport chain, potentially reducing electron leakage and contributing to the mitigation of ROS accumulation caused by salt stress–induced oxidative stress that impairs mitochondrial function. In addition, the enhancement of SAM-dependent methyltransferase and ligase activities might suggest epigenetic regulation and enhanced translational fidelity involved in the hypersaline survival strategies (Fig. S4b and Table S12). Analogous strategies have been reported in plants, where DNA methylation and histone modifications contribute to stress tolerance by altering gene expression patterns [43].

Together with other possible mechanisms detected by the above techniques, these findings were integrated into a unified metabolic and regulatory pathway map (Fig. 3e and Table S12). To further validate the functions of DEGs, we performed RNAi-based knockdown and survival assays as proof-of-concept under different stress conditions. We functionally validated two top-ranking DEGs: evm.model.tig639.2 (unannotated), which showed a dramatic increase in expression under high salinity, and evm.model.tig2212.1 (protein kinase), which was strongly upregulated in response to high salinity, low temperature, and hypoxia (Fig. 3f, g, k–m, and Table S15). In addition, we performed targeted functional validation of three TreT paralogs that were repeatedly differentially expressed under distinct stressing conditions. The knockdown phenotypes of these TreT genes were consistent with their transcriptional expression patterns, confirming their contrasting roles in stress resilience (Fig. 3h–j, and Tables S15 and S16). Investigations into the precise mechanisms and pathways involving these genes are currently underway.

### TreT glycosyltranferases, horizontally transferred from an anaerobic bacterium, contribute to resilience against multiple stressors

Given the abundance of stress resilience genes in the genome of *Apourosomoida* sp., it is unlikely that all were inherited from a common ancestor with *N. apoaustralis*. We hypothesize that some of these genes originated from recent horizontal gene transfer (HGT) of prokaryotic genes, and are not commonly found in other ciliates. We then performed HGT analyses and functional validations of the candidate genes. Specifically, we applied a series of analyses and stringent filtering steps, including removal of contaminating bacterial sequences during genome assembly, Alien Index (AI) screening (AI >0.05) against the NCBI non-redundant (nr) protein database, phylogenetic analyses, the requirement that contigs harboring candidate genes possess telomeres at both ends, and support from multiple raw Nanopore reads spanning the entire chromosome (see Supplementary Materials for details). We further validated the genomic origin of candidate genes by inspecting Nanopore long reads to confirm the physical linkage between candidates and telomeric repeats, and by confirming that their sequencing coverage was consistent at both the DNA and RNA levels (Figs S4c and S5a). To minimize false positives arising from genes of *de novo* origin within the ciliate genome, we applied additional filtering criteria, including gene structure analysis (excluding candidate genes with introns; intronless genes are prevalent in ciliates, Table S3), differential expression under stress conditions, and homology searches across ciliates (excluding genes commonly present in ciliate lineages). We also excluded candidates detected in the genomes of nine phylogenetically diverse ciliates to rule out genes broadly distributed in ciliate lineages (Tables S17 and S18).

Eventually, we identified two genes acquired through HGT: TreT glycosyltransferase and L-threonine aldolase. Among them, TreT glycosyltransferase (evm.model.tig9057.1, abbreviated as TreT 9057.1; Table S17) is a known enzyme that synthesizes α,α-trehalose, a universal stress-protective biomolecule. Its presence was also supported by our Raman, HRESIMS, and differential gene expression results (Figs. 1j and l, 3c and d, and Tables S12–S14). To rigorously exclude bacterial contamination and verify the endogenous nature of the TreT candidate, we performed a detailed structural validation (Fig. S5a). The contig (contig9057) harboring TreT 9057.1 is physically flanked by ciliate telomeric repeats (C_4_A_4_)_n_, and high-quality Nanopore reads (mean quality score 16.40) spanning the entire chromosome confirmed this linkage, supported by synchronized sequencing depth of coverages at both DNA and RNA levels (Fig. S5a). To evaluate the divergence of the candidate TreT genes from their homologs in prokaryotes and fungi, we conducted sequence identity analysis (Fig. S5b). The analysis revealed that the ciliate genes shared 24.0%–42.9% sequence identity with bacterial/archaeal homologs, whereas their identity with fungal homologs was substantially lower (20.6%–28.9%), indicating moderate divergence from bacterial/archaeal homologs and supporting their HGT origin rather than vertical inheritance from fungal or other eukaryotic lineages.

**Figure 4.**
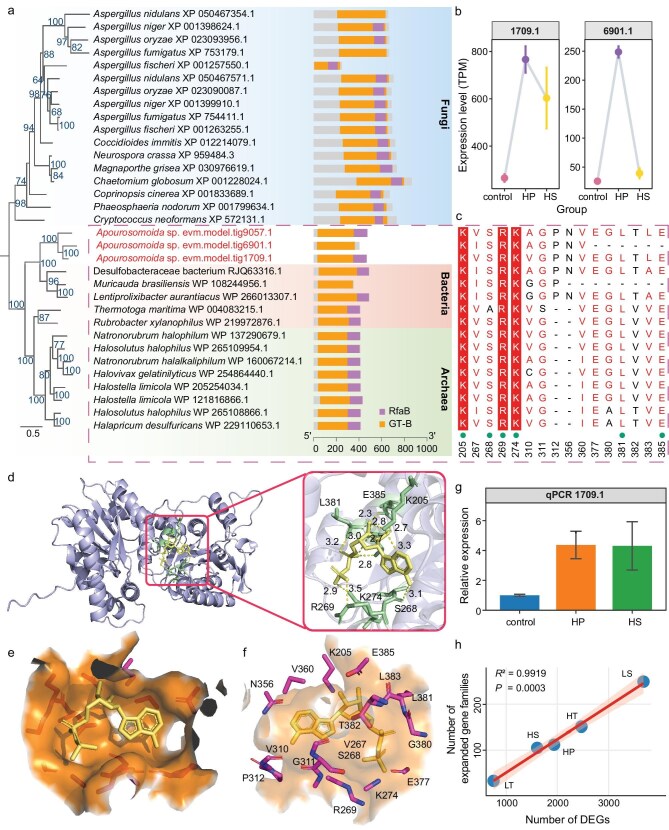
HGT analysis and functional validation of the TreT genes in *Apourosomoida* sp. (a) Among 166 species analyzed, only 33 homologs were identified as members of the TreT gene family and included in the phylogenetic analysis, with conserved domain analysis (right; RfaB and GT-B are domains for GT4 trehalose phosphorylase). Bootstrap values below 50% are not displayed. (b) Differential expression of TreT paralogs (1709.1 and 6901.1) under hypoxia (0.5 mg/L) and high salinity (80 PSU). (c) Amino-acid alignment near the ligand-binding site (5 Å) of TreT 1709.1 protein, green dots mark the hydrogen-bond sites mediating TreT-ADP binding. (d) Molecular docking of TreT 1709.1 with ADP ligand; the crucial amino acids are visualized in green sticks; the ADP molecule in yellow sticks; the hydrogen bonds in yellow dashed lines. (e and f) Structural details of the ADP-binding pocket (5 Å radius), the crucial amino acids are shown in sticks, highlighted in magenta. (g) RT-qPCR validation of TreT 1709.1 expression under hypoxia and high salinity. (h) Linear regression illustrating a positive correlation (*R*^2^ = 0.9919, *P* = 0.0003) between the number of DEGs and the number of expanded gene families containing some of them, across five stressor treatments: LT: low temperature (4°C), HT: high temperature (28°C), HP: hypoxia (0.5 mg/L), LS: low salinity (20 PSU), HS: high salinity (80 PSU).

TreT 9057.1 clusters with a glycosyltransferase from a Desulfobacteraceae bacterium, with an estimated divergence time of ∼62.15 million years (Fig. 4a and Fig. S5b). Members of the Desulfobacteraceae family are also known to be strictly anaerobic and commonly inhabit hypersaline waters, including salt lakes [44]. This suggests that TreT 9057.1 was likely acquired through an HGT event from a Desulfobacteraceae bacterium. Subsequently, gene duplications gave rise to two additional TreT paralogs in the *Apourosomoida* sp. genome, TreT 1709.1 and TreT 6901.1 (Fig. 4a). To note, these TreT genes appear to have undergone functional divergence under different environmental conditions. According to DEG analyses (Tables S12–S14), TreT 1709.1 was significantly up-regulated under both hypoxic and hypersaline conditions, whereas TreT 6901.1 was significantly up-regulated only under hypoxic conditions. In contrast, the original HGT copy TreT 9057.1 was expressed at low levels under all treatments and did not display any significant differential expression under any condition.

Based on the above results, we chose TreT 1709.1 for further structural and functional investigation. Conserved domain search analysis based on the primary sequence explicitly identified two distinct domains [45]: the RfaB domain, which is typically involved in the biosynthesis of extracellular polysaccharides (EPS) and the core region of lipopolysaccharides (LPS); and the GT-B domain, which catalyzes the reversible synthesis of α,α-trehalose from nucleoside diphosphate glucose and glucose (Fig. 4a). Both domains are highly conserved in bacteria and fungi (Fig. 4a). Furthermore, TreT 6901.1 lacks the RfaB domain, consistent with the aforementioned subfunctionalization (Fig. 4a and c). Structural prediction via AlphaFold3 revealed a high-confidence binding model (ipTM = 0.91, Ptm = 0.91) between TreT 1709.1 and ADP, a necessary interaction involved in trehalose synthesis. The ligand-binding pocket functions as a catalytic scaffold that positions ADP for nucleotidyl transfer. Within this functionally essential 5-Å radius of the predicted ADP-binding site, eight highly conserved residues (K205, S268, R269, K274, G311, E377, L381, E385) were identified as putative mechanistic determinants (Fig. 4c–f). Among them, K205, S268, R269, K274, L381, and E385 form critical hydrogen bonds with ADP, suggesting their importance in maintaining ligand-binding stability (Fig. 4c and d). Notably, T382 near the binding site shows substantial sequence divergence across this protein family, potentially associated with substrate specificity or ecological niche-related regulatory functions. Additionally, a highly conserved GGGVAE motif (residues 39–44; Fig. S5c) was identified within the sequence, which is characteristic of the nucleotide-recognition domain in GT-B glycosyltransferases. This motif has been previously implicated in UDP-glucose binding during trehalose biosynthesis, supporting evolutionary retention of an ancestral catalytic architecture at the 3D-structure level [46].

**Figure 5.**
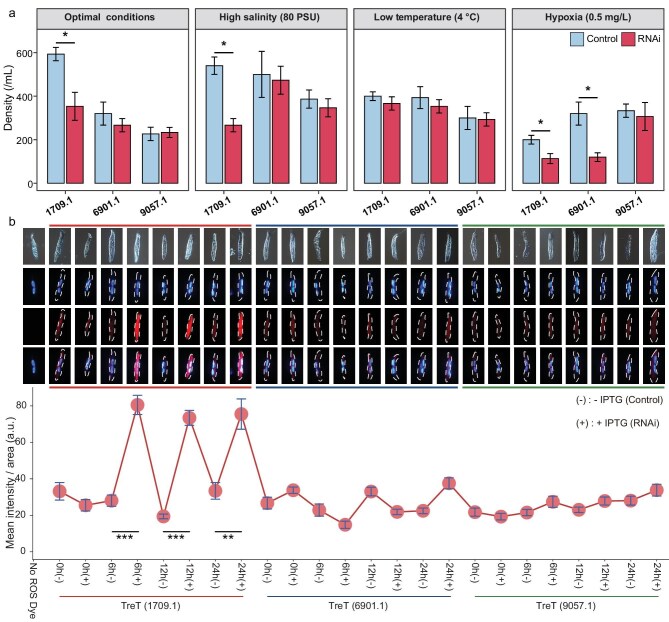
Assessment of cell survival and oxidative stress levels following RNAi-mediated knockdown of three TreT paralogs. (a) Cell density of *Apourosomoida* sp. following the silencing of TreT 1709.1, TreT 6901.1, and TreT 9057.1 under four different conditions: optimal conditions (50 PSU, 16°C), high salinity (80 PSU), low temperature (4°C), and hypoxia (0.5 mg/L). Blue bars represent the non-induced control groups (Control), and red bars represent the IPTG-induced RNAi groups (RNAi). (b) Detection and quantification of intracellular reactive oxygen species (ROS) under optimal conditions. Top panels: representative fluorescence microscopy images of cells stained with Hoechst 33342 (blue, nuclei) and dihydroethidium (DHE; red, ROS). Colored frame outlines correspond to the group colors shown in the bottom panel. Bottom panel: quantitative analysis of mean ROS fluorescence intensity normalized to cell area across different time points (0, 6, 12, and 24 h) post-induction. Note that the x-axis labels are aligned with the figure columns in the top panels. Comparisons were made between non-induced groups [(−): −IPTG] and induced groups [(+): +IPTG]. The ‘No ROS Dye’ condition served as a negative control for autofluorescence. Data are presented as mean ± SD of three biological replicates. Statistical significance was determined using a two-tailed Student’s *t*-test (*P < 0.05, **P < 0.01, ***P < 0.001).

We further validated the expression pattern of TreT 1709.1 by RT-qPCR (Fig. 4g and Table S16). Subsequent RNAi-mediated knockdown then revealed that TreT 1709.1 plays a critical role in cell survival under both hypoxic and hypersaline stress conditions, suggesting its essential role in stress resilience (Figs 3g, 5a, and Table S16). Notably, silencing TreT 1709.1 also significantly reduced cell survival under optimal conditions (50 PSU, 16°C), a phenotype that aligns with the substantial accumulation of intracellular ROS observed in the knockdown group under the same conditions (Fig. 5a and b). From a bioenergetic perspective, maintaining the constitutive expression of a gene imposes a continuous metabolic cost; if such a gene were functional only under sporadic stress conditions, this expenditure would be energetically inefficient. Therefore, the high basal lethality and oxidative stress observed upon knockdown suggest that TreT 1709.1 is not merely a stress-responsive reserve but has been assimilated into the core metabolic network to mitigate basal oxidative stress, thereby justifying the metabolic cost of its constitutive maintenance.

In contrast to the broad essentiality of TreT 1709.1, TreT 6901.1 appears to have undergone functional specialization. Consistent with its transcriptomic profile—which showed upregulation specifically under low oxygen—RNAi-mediated silencing (knockdown efficiency: ∼81.1%) significantly impaired cell survival exclusively under hypoxic conditions, with no observable defects under high salinity or optimal conditions (Figs 3i and 5a, and Table S16). Finally, the ancestral-like copy TreT 9057.1 displayed signs of limited physiological contribution. Despite achieving a knockdown efficiency of ∼71.9% (Fig. 3j), its silencing did not result in significant changes in cell survival under any of the tested conditions, which is in alignment with its consistently low expression levels. Collectively, these findings illustrate the functional divergence of the TreT paralogs: a broad stress response in TreT 1709.1, hypoxia-specific specialization in TreT 6901.1, and limited responsiveness in TreT 9057.1.

### Evolutionary dynamics of genes associated with the polyextremophilic capabilities

To elucidate the evolutionary history of the stress-associated genes, we first performed analyses of gene-family expansion and contraction, using published genomes of eight ciliates and two Excavata species, as well as those of *Apourosomoida* sp. and *N. apoaustralis* assembled in this study.

In total, the 12 species comprise 364565 genes, with 315948 assigned to 46356 gene families, with 584 gene families common to all species. Seven single-copy orthologous genes used for divergence-time analysis, using calibration points from Timetree, suggest that *Apourosomoida* sp. diverged from its common ancestor with *N. apoaustralis* ∼424 million years ago (Fig. 3a). It is not uncommon for even congeners of ciliates to diverge from each other for hundreds of millions of years in ciliates, which are known to have an extremely long evolutionary history [47,48]. A total of 289 expanded gene families and 801 contracted gene families were identified in *Apourosomoida* sp. (Fig. 3a). The expanded gene families are enriched in biological processes such as DNA repair, DNA damage response, and the ubiquitin-dependent protein catabolic process, suggesting a potential enhancement in the targeted degradation of damaged or misfolded proteins (Fig. S6a and Table S19). The number of DEGs in response to various stressors showed a positive correlatation with the number of expanded gene families that include some of the DEGs (*R*^2^ = 0.9919, *P* = 0.0003; Fig. 4h). Additionally, among the five spirotrich species, there are 4436 shared homologous gene groups between *Apourosomoida* sp. and *N. apoaustralis*, with 168 not present in any of the other three spirotrich species (Fig. 3a). Specifically, *Apourosomoida* sp. has 350 unique gene families not present in any other ciliates, potentially reflecting lineage-specific genomic innovations (Fig. S6b). These unique gene families are especially enriched in biological processes associated with efficient resource utilization and cell cycle regulation (Fig. S6c and Table S19). For example, enhanced autophagy pathways may facilitate the recycling of cellular components during nutrient scarcity, while enrichment in lipid transport pathways is consistent with a role in maintaining membrane fluidity and osmotic balance under high-salinity conditions. Moreover, refined cell cycle regulation might contribute to rapid proliferation during transiently favorable conditions and energy-efficient dormancy during prolonged stress. Together, these genomic features may collectively support the organism’s capacity to survive and thrive in the extreme environments of the Tibetan salt lake.

We then estimated selective pressures on homologous gene pairs between *Apourosomoida* sp. and *N. apoaustralis*. In >99% of genes, the dS values (rate of synonymous mutations) exceeded one, indicating widespread mutation saturation possibly due to the extremely long divergence time between the two species—despite *N. apoaustralis* being the closest culturable relative with relatively high genome assembly quality. Given that such saturation renders the dN/dS ratio unreliable and in principle all genes within a species should have similar dS, we instead focused on the 20 genes with the highest dN values (rate of nonsynonymous mutations), all of which were also differentially expressed under at least one of the stressing conditions tested in this study (Table S20). Most of these genes, possibly under natural selection, are involved in mechanisms potentially linked to stress resilience, such as phosphorelay signal transduction, which may enhance sensing sensitivity to salinity fluctuations and cold stress, dsDNA-binding proteins implicated in maintaining genetic fidelity under high ionic strength, mitotic CDK holoenzyme complex regulators optimizing cell cycle checkpoint control for energy conservation in hypoxia, and perinuclear-localized gene products hypothesized to form stress-response hubs to accelerate environmental signal relay to the nucleus (Fig. S7 and Table S19). Future exploration, particularly with population-level data, will provide more insights into the evolutionary mechanisms of stress-resilience in *Apourosomoida* sp.

## DISCUSSION

Using an integrative approach combining cell biology, systematics, biochemistry, multi-omics, molecular genetics, and evolution analyses, we explored the polyextremophilic capabilities of a rare ciliate *Apourosomoida* sp. LHA081A01 from a Tibetan salt lake. Our findings reveal numerous stress-resilience mechanisms enabling survival under low temperature, high salinity, and hypoxic conditions—most notably, the accumulation of trehalose, which likely plays a key protective role. Additionally, horizontal gene transfer and widespread natural selection were identified in key stress-resilience genes, many of which are differentially expressed under specific stressors and belong to expanded gene families.

Nevertheless, further functional studies are needed to fully uncover the polyextremophilic capacities, particularly on stress-associated genes lacking functional annotations and unidentified biomolecules abundant in the cells (Figs 1k and l, 3g, and Table S15). Besides, laboratory stress treatments cannot fully replicate the complexity of natural environments, such as the influence of air pressure, seasonal variations, and microbial interactions, which are crucial in shaping microbial traits in high-altitude ecosystems. Future studies could apply long-term *in situ* monitoring to better capture the mechanisms under natural conditions. Furthermore, this study is based on observations from a single isolate, which may limit the generalizability of our findings. Expanding sampling efforts to include a broader range of salt lakes—despite several of our recent unsuccessful attempts—and conducting population-level analyses would help address this limitation.

Although *Apourosomoida* sp. has an extraordinary toolbox to tackle the environmental challenges of the Tibetan salt lake, this does not guarantee its success in the face of climate change. The salinity of many Tibetan salt lakes is decreasing due to a regional warming rate three times faster than the global average, which accelerates glacial melt. This trend is further intensified by shifts in the balance between precipitation and evaporation [49,50]. Correspondingly, the survival rate of *Apourosomoida* sp. decreases under high temperature or low salinity (Fig. 1n and o). Differential gene expression analyses at an elevated temperature (28°C) or reduced salinity (20 PSU) further confirm the severe threat to its viability, as indicated by the enrichment of autophagy, double-strand break repair, DNA damage response, and so on (Fig. S8a and c, and Tables S12, S21, and S22). In low-salinity environments, the significant enrichment of cytoskeletal and microtubule motor activity is consistent with the observed transformation of cells into a spherical shape. Additionally, the induction of oxidoreductase activity and FAD-binding proteins further indicates cellular mechanisms aimed at mitigating the accumulation of ROS, which may arise as byproducts of disrupted cellular homeostasis (Fig. S8b and d, and Table S12). This thus provides a vivid example of how climate change might affect protozoa in high-altitude salt lakes.

Across all tested environmental stressors (LT: low temperature, HT: high temperature, HP: hypoxia, LS: low salinity, HS: high salinity), transcriptomic analyses identified 35 shared DEGs significantly enriched in pathways potentially contributing to resilience against multiple stressors (Fig. S9a). Among them, the RNA helicase 9617.1, by dynamically remodeling RNA secondary structures and RNA-protein complexes, safeguards the fidelity and efficiency of gene expression (Fig. S3a–f). The Camp-dependent protein kinase complex acts as a central signaling hub, integrating temperature and salinity stresses through phosphorylation cascades in order to regulate downstream cellular responses. Differential expression of cytoskeleton-related genes suggests dynamic structural adjustments for osmoregulation in hypersaline conditions, potentially mediated by phosphorylation modifications. Coordinated activation of lipid metabolism and autophagy pathways implies functional coupling between energy supply and transmembrane transport demands. Concurrent translation suppression reflects the necessity of protein quality control systems under combined stresses (Fig. S9b). This hierarchical interaction network thus reveals a survival strategy where phosphorylation core nodes coordinate cellular plasticity, metabolic reprogramming, and stress responses, supporting the success of *Apourosomoida* sp. in habitats characterized by hypersalinity, low dissolved oxygen, and seasonal freeze–thaw cycles.

Although *in situ* measurements from Yibug Caka indicate non-extreme dissolved oxygen levels (DO 5.36–6.64 mg/L in both summer and winter), which appear inconsistent with the strong hypoxia resilience of *Apourosomoida* sp., this species is predominantly periphytic, inhabiting the sediment–water interface and even the sediment itself. Such microhabitats, particularly when combined with localized organic inputs (e.g. from wildlife), can generate transient or spatially restricted hypoxic zones that are not captured by bulk water measurements. Alternatively, hypoxia tolerance in this species may be maintained as a ‘hitchhiking’ trait, co-selected alongside high-salinity tolerance due to the pleiotropic effects of TreT.

The genome size and protein-coding gene number, both among the lowest recorded in ciliates (Table S3), as well as the majority of genes having no introns, also possibly facilitate survival under harsh environmental conditions by minimizing energy costs and optimizing genes associated with DNA replication, maintenance, and transcription. This aligns with the extremophilic ciliate *Fabrea salina*, which has the smallest known ciliate genome and tolerates salinities ranging from 35–180 PSU (Table S3) [16], suggesting possible convergent evolution driven by similar ecological constraints. Notably, unlike *Apourosomoida* sp., *F. salina* lacks trehalose biosynthesis enzymes and instead utilizes betaine synthesis for osmoprotection, further reflecting ciliates’ diverse strategies to tackle hypersaline environments. Using the AI index method, we investigated HGT events in this species and identified ten genes likely acquired from bacteria. However, most of these represent ancient HGT events, as 90% of the lineages carrying the candidate genes belong to Heterotrichida ciliates, suggesting that the transfers likely occurred in the common ancestor of this group.

The evolutionary origin of *Apourosomoida* sp. dates back ∼424 million years to the Early Paleozoic era, when it diverged from its common ancestor shared with *N. apoaustralis* (Fig. 3a). Throughout its geological history, the Paleo-Tethys Ocean region corresponding to the present-day Tibetan Plateau underwent multiple dramatic environmental changes. Notably, during the Late Devonian period (419 to 359 Mya), the Earth experienced a significant global environmental crisis characterized by widespread oceanic anoxia and mass species extinction, known as the Late Devonian extinction event [51–53]. Although the divergence of *Apourosomoida* sp. slightly predates this event, the prolonged environmental fluctuations in the Paleo-Tethys Ocean region may have had profound effects on the evolutionary trajectory of its ancestor [54]. These environmental upheavals, together with the later continuous rises of the Tibetan Plateau, possibly drove ancestral populations of *Apourosomoida* to evolve diverse mechanisms against extreme stresses, such as low oxygen, high salinity, and low temperatures, ultimately shaping their polyextremophilic capabilities observed today. While our comparative genomic analyses identified candidate genes revealing high divergence and transcriptional responsiveness, we acknowledge that conclusively distinguishing positive selection from relaxed purifying selection remains challenging in the absence of population-level data. Acquiring such comprehensive datasets is currently limited by substantial logistical challenges associated with the Tibetan high-altitude salt lakes, where extreme remoteness and harsh conditions make extensive sampling exceptionally difficult. Future studies should aim to overcome these constraints to further clarify the evolutionary forces shaping *Apourosomoida* sp.

In conclusion, this study provides novel insights into the stress-resilience capabilities of *Apourosomoida* sp. in the extreme environment of the Tibetan salt lakes. Through integrative analyses, we reveal that this rare ciliate has polyextremophilic features driven by a combination of horizontal gene transfer, natural selection, and gene-expression plasticity. The species demonstrates an extraordinary capacity to endure high salinity, hypoxia, and low temperature, facilitated by protective metabolites such as trehalose and β-carotene, robust antioxidant systems, and dynamic regulation of stress-response pathways. The unique gene-sized chromosome, relatively high GC content, and minimized intronic load further suggest that genome architecture might contribute to long-term survival in harsh conditions. Nevertheless, the existence of this ciliate is still challenged by climate change, due to its inability to withstand high temperature and low salinity. This study demonstrates high-altitude microbial eukaryotes as valuable extremophile models for studying stress-resilient mechanisms and evolution.

## MATERIALS AND METHODS

We quantified the polyextremophilic capabilities of *Apourosomoida* sp. LHA081A01, collected from Yibug Caka, a salt lake in Tibet (32.99°N, 86.66°E; altitude 4533 m) on 1 August 2020. Cell survival was evaluated across gradients of salinity, dissolved oxygen, and temperature. Raman spectroscopy, HPLC, and HRESIMS analyses were performed to identify cellular biomolecules and sugars. *De novo* assembly of the macronuclear genome was carried out using Oxford Nanopore long-read and Illumina NovaSeq 6000 PE150 sequencing and Canu, followed by annotation with AUGUSTUS, EuGene, Omicsbox, and transcriptomic data. Differential gene expression and pathway enrichment analyses were performed under varying environmental conditions. Evolutionary investigations included analyses of horizontal gene transfer, dN/dS, gene family expansion/contraction, and phylogenomics. Structural and functional validation of candidate genes was done using AlphaFold3, RT-qPCR, and RNAi, complemented by survival assays under treatment and control conditions. Detailed procedures, parameters, and all relevant references pertaining to the materials, reagents, instruments, and analytical approaches described herein are provided in the Supplementary Materials.

### Resource availability

All raw sequences in this research are publicly available at National Genomics Data Center (NGDC, https://www.cncb.ac.cn/), China National Center for Bioinformation, under the project accession numbers CRA026007, CRA026008. Two newly assembled and annotated macronuclear genomes are uploaded to the NGDC: GWHGEEQ00000000 (*Apourosomoida* sp. LHA081A01) and GWHGEEPO0000000 (*Notohymena apoaustralis* SC0818). Strains are available upon request. All scripts for data analyses are available at: https://github.com/IEMB-LEG/Lab-of-Evolutionary-Genomics. All data needed to evaluate the conclusions in the paper are present in the paper and/or the Supplementary Materials.

## Supplementary Material

nwag117_Supplemental_Files
