# Supplementary material for: Cross-domain transfer of trehalose biosynthesis genes contributes to adaptation in high-altitude environments
Source: Natl Sci Rev. 2026 Feb 25;13(8):nwag117. doi: 10.1093/nsr/nwag117 (PMC13166879; doi:10.1093/nsr/nwag117)
Supplement: nwag117_Supplemental_Files [file nwag117_supplemental_files.zip › Supplementary Materials-0825-NSR-re260125.docx]

**Supplementary Materials**

**Species isolation, culture and identification**

*Apourosomoida* sp. LHA081A01 and its freshwater relative *Notohymena apoaustralis* SC0818 were collected from two distinct environments. *Apourosomoida* sp. was collected from the salt lake Yibug Caka of Tibet on August 1, 2020 (32.99 °N, 86.66 °E; altitude 4533 m; 16.32 °C; dissolved oxygen 5.36 mg/L; salinity 49.85 PSU; pH 8.76). *Notohymena apoaustralis* was collected from a freshwater pond in Qingdao, Shandong Province on August 18, 2004 (120.35 °N, 36.07 °E; altitude 29 m; temperature 26.63 °C; dissolved oxygen 10.92 mg/L; salinity 0 PSU; pH 7.69; a gift from Lab of Protozoology, Ocean University of China) (1). The strains were fed with the bacteria *Escherichia coli* MG1655, which were suspended in either sterilized salt water (for *Apourosomoida* sp.; Instant Ocean aquarium salt, Cat. No. 216034) or sterilized DI water (for *N. apoaustralis*) at an OD600 of 0.2. Cultures were maintained at 16 or 24 °C. Species identification was performed based on morphological features *in vivo* and after silver impregnation, and 18S rRNA gene sequences (*Apourosomoida* sp. LHA081A01, NCBI GenBank accession number: PV626721.1). The PCR primers used were EukA (5'-AACCTGGTTGATCCTGCCAGT-3') and EukB (5'-TGATCCTTCTGCAGGTTCACCTAC-3'). 2× Hieff Canace^®^ AdvanceFast PCR Master Mix (With Dye; Yeasen Cat. No. 10164ES03) was used for gene amplification. Living cells of *Apourosomoida* sp. were observed and photographed using Nikon NI-U and Olympus SZX16 microscopes with bright field and differential interference contrast modes. To reveal the infraciliature and nuclear apparatuses, cells were stained with protargol following the method of Wilbert (1975) (2) and Hoechst 33342.

For survival assays, in the temperature treatments, groups of organisms (10 individuals per group, with 4 replicates per temperature level) were exposed to a wide gradient ranging from 4 °C to 28 °C in 4 °C increments. Each group was placed on a glass slide containing 200 μL of saline solution (50 PSU; Instant Ocean aquarium salt; Cat. No. 216034) and incubated for 1 hour before survival assessment. In the salinity treatment, a wide gradient of 0–150 PSU was tested (4 replicates per salinity level): 10 organisms were placed on glass slides with artificial saline solutions of varying salinities. To prevent evaporation, each slide was transferred to a 90 mm-diameter Petri dish with thin layer of water and sealed, followed by counting cells on a dissection microscope after 1 hour. For hypoxia tolerance experiments, we followed a classic anaerobic ciliate culture protocol (3), organisms were incubated in 15 mL of 50 PSU saline solution within 90 mm-diameter Petri dishes placed in an anaerobic jar for 24 hours in 16 °C (4 replicates; dissolved oxygen levels were measured with Universal Multi-Parameter Handheld and Optical IDS dissolved oxygen sensors; Cat. No. Multi 3320 and FDO^®^ 925, WTW) and maintained below 0.5 mg/L (4). Survival under hypoxic stress was determined by comparing pre- and post-treatment cell densities.

**Raman assays**

Raman spectroscopy of *Apourosomoida* sp. was done by measuring five points of the cell surface of each cell and in total 20 replicate cells. Briefly, after 10 days of culturing, the cells were enriched by centrifugation (1000 g, 5 min) twice in 15 mL centrifuge tubes. Paraformaldehyde fixative (Servicebio, Cat. No. G1101) was first diluted with sterilized saline solution at a 1:3 ratio, and the fixative was added to the sample at a 1:5 ratio, with fixation lasting for 15 minutes. The subsequent procedures were conducted on a Raman spectrometer (FlowRACS, Qingdao Single-Cell Biotechnology, Qingdao, China). Automated Raman spectrum collection was performed using RamLIS for acquisition feedback and analyzed using RamEX. Five spectra were collected for each cell, with 20 individual cells sampled in total. The complete spectral data (400–3050 cm^-^¹) of multiple single cells per sample were used to build a database, followed by min-max normalization of the Raman spectral intensity for standardization. The normalized Raman spectra (500–2000 cm⁻¹) were averaged using LabSpec 6 software to obtain a single representative mean spectrum for visualization. Raman spectroscopy identified characteristic peaks at 1,520, 1,160, and 1,006 cm^-1^ for β-carotene (5) and around 1,130 cm^-1^ for trehalose (Normalized Intensity 0.35 a.u.) (6, 7), confirming their intracellular accumulation.

**HPLC &** **HRESIMS assays**

*Apourosomoida* sp. was mass cultured to a density of approximately 2000/mL. Cells were harvested by centrifugation at 5000 g for 10 minutes. The collected biomass was used for carotenoid extraction and subsequent HPLC&MS analysis. High-performance liquid chromatography (HPLC) analysis of the compounds was performed using an Agilent Infinity II 1260 high-performance liquid chromatograph with a 5-AmyCoat column (4.6 mm × 250 mm, 5 μm; Kromasil^®^, Cat. No. K08971226). The mobile phase consisted of n-hexane (Fisher Chemical, CAS. 110-54-3) and isopropanol (Fisher Chemical, CAS. 67-63-0), with an isocratic elution using 5% isopropano. The flow rate was set at 1.0 mL/min, and the column temperature was maintained at 30 °C. Detection was carried out at 450 nm, with an injection volume of 10 μL. A total of 0.45 mg of β-carotene (Cat. No. K08971226) standard was accurately weighed and dissolved in 1.5 mL of a chloroform–isopropanol (1:1, v/v) mixture. Then, 100 μL of this stock solution was diluted to 20 mL with the same solvent mixture to obtain a final concentration of 1.5 μg/mL. The solution was filtered through a 0.22 μm microporous membrane prior to use. The lyophilized ciliate sample was dissolved in 2 mL of chloroform–isopropanol (1:1, v/v) mixture and sonicated for 1 hour at 25 °C (100 W, 40 kHz). The solution was filtered through a 0.22 μm microporous membrane and used directly for HPLC analysis.

For mass spectrometry (MS) analysis, two separate extraction protocols were applied to accommodate positive- and negative-ion modes. For positive-ion mode detection, lyophilized biomass was extracted with 1.5 mL of HPLC-grade isopropanol:chloroform (3:1, v/v), followed by sonication at 25 °C for 1 hour (100 W, 40 kHz) and centrifugation. The resulting supernatant was filtered through a 0.22 μm microporous membrane to yield sample solution 1. For negative-ion mode analysis, a separate portion of the lyophilized sample was extracted with 1.5 mL of HPLC-grade methanol (XiLONG Science, CAS. 67-56-1): water (3:1, v/v) using identical sonication and filtration conditions, resulting in sample solution 2. Both solutions were subjected to high-resolution mass spectrometric analysis using a Micromass Q-TOF instrument equipped with an electrospray ionization (ESI) source.

**DNA and RNA extraction**

Single cells of each strain were first isolated, cultured in wells of 6-well plates for 48 hours, and then mass cultured in 1 L *E. coli* suspension with OD600 of 0.3. The cultures were incubated at 16 °C (*Apourosomoida* sp. LHA081A01) and 24 °C (*N. apoaustralis* SC0818). To reduce bacterial contamination, cell culture was filtered through 15 µm sterile filter papers with a Milliflex Oasis^®^ filtration pump for EZ-Fit^®^ Filtration Unit. Genomic DNA and total RNA was extracted using the MasterPure^®^ Complete DNA & RNA Purification Kit (Cat. No. MC85200; Lucigen, USA), followed by further purification with the Genomic DNA Clean & Concentrator™-10 Kit (Cat. No. ZRC000496; ZYMO).

**Genome and transcriptome sequencing**

For Oxford Nanopore long-read sequencing, the libraries of *Apourosomoida* sp. LHA081A01 and *N. apoaustralis* SC0818 were prepared using the ligation sequencing kit (SQK-LSK114; Nanopore) and loaded into R10.4.1 flow cells. The SC0818 library was sequenced on a MinION machine in our lab, while the LHA081A01 library was sequenced on a PromethION platform at NextOmics Biosciences (Wuhan, China).

For Illumina sequencing, DNA libraries were prepared using the TruePrep^®^ DNA Library Prep Kit V2 for Illumina (Cat. No. TD501-01; Vazyme) and the TruePrep^®^ Index Kit V2 for Illumina (Cat. No. TD202; Vazyme). We followed a miniaturized protocol developed by our lab, which uses approximately one-sixth of the reaction system typically used by service providers (8). The concentration and quality of the DNA samples were measured using a Qubit 3.0 (Thermo Fisher Scientific, US) and a Nano-300 Micro-Spectrophotometer (ALLSHENG, China).

For size selection of the sequencing libraries, we followed the manufacturer’s instructions and used the E.Z.N.A.^®^ Gel Extraction Kit (Cat. No. D2500-02; OMEGA). The prepared libraries were then sequenced on a NovaSeq 6000 platform at Novogene (Beijing, China). RNA libraries were prepared using the NEBNext^®^ Ultra^TM^ RNA Library Prep Kit (Cat. No. E7370L).

**Macronuclear genome assembly**

The Illumina raw reads were filtered using fastp v0.23.2 with default parameters (9), while the Nanopore raw reads were preliminarily filtered using NanoFilt v2.7.1 with the parameters -q 7 -l 500 --headcrop 50 --tailcrop 50. We obtained 16.67 Gbp of Nanopore clean reads for *Apourosomoida* sp. (mean read length: 2,679 bp; sequencing coverage: ~373×) and 4.19 Gbp for *N. apoaustralis* (mean read length: 4,958 bp; sequencing coverage: ~41×) (10), alongside 5.88 Gbp and 25.80 Gbp of Illumina NovaSeq 6000 clean reads, respectively. The clean reads were then aligned to the *E. coli* genome (GCA_000005845.2) using bwa v0.7.17 and Samtools v1.1.0, and any reads with a total alignment > 80% of their own lengths were considered as contaminated reads and removed (11, 12).

The genome assembly of the Nanopore reads was performed using Canu v2.1.1 (13). After the initial assembly, three rounds of genome polishing were conducted using Racon v1.4.3 and Pilon v1.2.4 (14, 15). To minimize the presence of bacterial sequences, contigs were filtered using blastn v2.9.0 (e-value: 1e-5) against a bacterial database (16), downloaded from NCBI (ftp://ftp.ncbi.nlm.nih.gov/genomes/refseq/bacteria/, on 19 June 2021), removing any contigs with ≥ 80% identity and a cumulative hit length ≥ 60%.

Redundancy in the draft genomes was reduced using purge_dups v1.2.6 twice (17). Additional contigs with > 45% GC content were filtered out, while these contigs were also examined for the presence of telomeric repeats or 18S rRNA gene sequences identified using RNAmmer v0.1.2 (18), in order to avoid the inadvertent removal of genuine genomic sequences. The final genomes of the two ciliates were assessed for quality using BUSCO v5.2.2 and QUAST v5.2.0 (19, 20).

**Gene prediction and annotation**

The repetitive regions in the genomes were first annotated and masked using RepeatMasker v4.1.0 and RepeatModeler v2.0.1 (21, 22). tRNAs were identified with tRNAscan-SE v2.0.9 (23). rRNA genes were searched using RNAmmer with the setting -S euk. Codon usage in the genomes was predicted using codetta v2.0 (24). RNA reads for structural annotation were first filtered with fastp and then aligned and converted with Hisat2 v2.1.0 (25) and Samtools. Genome-guided assembly was performed with Trinity v2.21.0 (--genome_guided_bam --genome_guided_max_intron 10000) (26). Structural prediction was carried out with augustus v3.3.3 (27), using the *Tetrahymena* model. A Weight Array Matrix (WAM) model was constructed using the *Apourosomoida* sp. genome and Trinity results, along with est data and protein sequences of *Oxytricha* downloaded from NCBI, as input files for EuGene v1.6.5 annotation (28). The results from Augustus and EuGene were integrated using EVidenceModeler v 2.0.0 (--min_intron_length 15 --stop_codons TGA). The final gene structures were predicted and annotated protein sequences were run in BUSCO to estimate genome completeness (29).

For *Apourosomoida* sp., the annotation results from EVidenceModeler (EuGene 5; Augustus 10) were used, while for *N. apoaustralis*, the results from EuGene were used. Protein-coding genes were annotated using the non-redundant protein database (nr) by blastp v 2.9.0 (e-value 1e-5 -word_size 3 -num_alignments 20 -max_hsps 20 -show_gis). Gene names were derived from the best hits. GO annotation was done with OmicsBox v1.4.11, and KEGG annotation was performed using the eggNOG-mapper v2 (30, 31).

The mitochondrial contigs were confirmed by blastn v2.9.0 alignment against the mitochondrial genome of *Oxytricha trifallax* downloaded from NCBI (GenBank: JN383843.1). CDS, tRNA, and rRNA predictions were performed using GeSeq (https://chlorobox.mpimp-golm.mpg.de/geseq.html) with added reference sequences from *Tetrahymena* and *Paramecium*, as well as the *Oxytricha trifallax* mitochondrial genome (32). 11 meiosis-related genes specific to Colpodea were retrieved from the UniProt database (https://www.uniprot.org/). These genes were subsequently used as queries for blastp searches against the annotated protein datasets of *Apourosomoida* sp. and *N. apoaustralis*. Based on the blastp results and gene annotations, the presence and distribution of meiosis-related genes were analyzed in both species.

**RNAseq-based gene expression analyses for different treatments**

Initially, saline solutions with salinities of 20, 50, and 80 PSU was prepared using Aquarium Systems^®^ Instant Ocean aquarium salt (Cat. No. 216034). The saline solution was sterilized and used for preparing *E. coli* suspension at an OD600 of 0.2, for each salinity. The ciliates were then subjected to different culture conditions as follows: LT-low temperature: 4 °C, 50 PSU; control: 16 °C, 50 PSU; HT-high temperature: 28 °C, 50 PSU; HP-hypoxia: 0.50 mg/L, 16 °C, 50 PSU; LS-low salinity: 16 °C, 20 PSU; HS-high salinity: 16 °C, 80 PSU; all groups had dissolved oxygen 6.87 mg/L, except the group HP—hypoxia group. The hypoxia condition (dissolved oxygen 0.50 mg/L) was achieved using 2.5 L anaerobic jars at 16 °C, 50 PSU (Cat. No. AG0025A, Oxoid AnaeroJar) with oxygen-scavenging chemicals (Cat. No. AN0025A; Thermo Scientific Oxoid AnaeroGen). Each condition was maintained for 24 hours. ~20 metabolically active cells per replicate under each experimental condition were lysed, and the resulting RNA was reverse transcribed into full-length cDNA, using the Single Cell Full Length mRNA-Amplification Kit (Cat. No.: N711; Vazyme). RNA libraries were then constructed from the cDNA using the TruePrep DNA Library Prep Kit V2 for Illumina (Cat. No.:TD503; Vazyme). The prepared libraries were then sequenced on a NovaSeq X-Plus platform at Novogene (Beijing, China). RNAseq clean reads were filtered using fastp, followed by alignment with Hisat2 and conversion with Samtools. Gene expression levels were calculated using StringTie v1.3.7 (setting: -e -B -G) and the prepDE.py3 script (33). Genes with significantly different expression levels were identified using DESeq2 v1.32.0 (|log_2_(fold change)| > 1 and *P*_adj_ < 0.05) (34). Gene Ontology (GO) analysis was performed using OmicsBox v1.4.11. GO pathway enrichment analyses of the significantly differentially expressed genes were conducted using clusterProfiler v4.0.2 (*P*_value_ < 0.05) (35). Pathway enrichment analysis using aPEAR v1.0.0 was also performed on the DEGs shared by all the five groups (36).

**Gene family and HGT analyses**

Gene family analysis was conducted using OrthoFinder v2.5.7 (37), on protein sequences from 12 high-quality genomes uploaded to NCBI or NGDC of China. To construct the species tree, 10 species within Alveolata (*Ichthyophthirius multifiliis* GCF_000220395.1, *Oxytricha trifallax* GCA_000295675.1, *Paramecium octaurelia* GCA_905182995.1, *Paramecium tetraurelia* GCF_000165425.1, *Stylonychia lemnae* GCA_000751175.1, *Tetrahymena thermophila* GCF_000189635.1, *Euplotes vannus* EVANNUS_28419 EvanDB, *Colpoda steinii* GWHERKZ00000000.1 National Genomics Data Center, *Apourosomoida* sp. GWHGEEQ00000000, *Notohymena apoaustralis* GWHGEEPO0000000), and outgroups (*Naegleria gruberi* GCF_000004985.1 and *Trichomonas vaginalis* GCF_026262505.1) were included. Seven single-copy orthologs (IF-2, MRP, NAE1, Rio1, EF-TU, ALG11, DIEXF) were aligned and concatenated using ParaAT v2.0 (38), and four-fold degenerate sites were extracted. IQ-TREE v3.0.1 was used to build the phylogenetic tree (-o Naegleria_gruberi -m MFP -nt AUTO -b 1000 ) (39). Divergence time was estimated using MCMCTree in PAML v4.9 (burnin=10,000, nsample=100,000, sampfreq=5) (40), with convergence verified. Calibration points were obtained from Timetree (http://www.timetree.org/; *Tetrahymena thermophila* vs. *Paramecium tetraurelia*; *Ichthyophthirius multifiliis* vs. *Tetrahymena thermophila*). To ensure the robustness of divergence time estimation, two independent runs were conducted, yielding highly congruent results. Gene family expansion and contraction analyses were performed using the CAFE v5.0.0 (41).

To calculate dN/dS values, we first identified orthologous genes between *Apourosomoida* sp. and *N. apoaustralis*. Protein sequence alignment was conducted using ParaAT v2.0 with parameters -m clustalw2 -f axt -g -c 6, followed by codon alignment through mapping aligned protein sequences to corresponding DNA sequences. Estimates of nonsynonymous (dN) and synonymous (dS) substitution rates, along with their standard errors, were calculated for each ortholog pair using the yn00 program in PAML v4.9 (42). Codon alignments were formatted for PHYLIP input and analyzed via configured yn00.ctl files. The dN/dS ratio and its standard error were calculated with the following formulae (43), assuming no co-variance between dN and dS:

$$\text{ω }= \frac{\text{dN}}{\text{dS}}$$

$$\text{SE }\left( \text{ω} \right) = \omega\sqrt{\left( \frac{\text{SE(dN)}}{\text{dN}} \right)^{2} + \left( \frac{\text{SE(dS)}}{\text{dS}} \right)^{2}}$$

To identify stress-associated genes acquired through recent horizontal gene transfer (HGT) in *Apourosomoida* sp., we performed multiple analyses, referring to methodologies and criteria established in previous studies (44, 45). 1) blastp searches against the NCBI nr database (February 2024 release; https://ftp.ncbi.nlm.nih.gov/blast/db/FASTA) using an e-value threshold of 10^-10^. Custom python scripts processed blastp results (-outfmt 6) by: a) assigning taxonomic classifications via NCBI taxonomy database, b) categorizing hits into three lineages RECIPIENT (SAR supergroup), GROUP (eukaryotes), and OUTGROUP (non-eukaryotes). Key parameters including bbhO (best outgroup bit-score), bbhG (best non-recipient eukaryotic bit-score), and maxB (self-match bit-score) were extracted to calculate the Alien Index (AI = (bbhO/maxB) - (bbhG/maxB)). 2) Confirming eukaryotic genome origin of candidate genes was performed to rule out bacterial contamination. This involved: (i) requiring that contigs harboring the candidate genes contain telomeres; (ii) identifying Nanopore reads spanning the entire length of the chromosome harboring the candidate genes; and (iii) requiring consistent sequencing coverage distributions at both DNA and RNA levels. 3) Phylogenetic validation using IQ-Tree reconstructed maximum likelihood trees with manual topology inspection, analysis between *Apourosomoida* sp. and 165 species, including 97 prokaryotes and 68 eukaryotes (--model-joint NONREV -B 1000 -T AUTO), broadly sampled from across the Tree of Life (Table S17). To determine the evolutionary directionality, the resulting phylogenetic tree was rooted using the Minimal Ancestor Deviation (MAD) method implemented in MADroot (46, 47) (https://github.com/davidjamesbryant/MADroot). 4) Significant differential expression under hypoxia, high salinity, or low temperature. 5) Without introns, to avoid ancient HGT. 6) Absence in ciliate genomes. Pairwise amino acid identity values among TreT homologs were calculated using Blastp with an e-value cutoff of 10. For the visualization, sequence pairs yielding e-value greater than 10 were considered to lack significant similarity, and their identity values were consequently treated as missing data (blank) in the heatmap.

**Molecular docking and RNA interference (RNAi), RT-qPCR validation and RNAi efficiency assessment**

To structurally validate the role of TreT 1709.1 in trehalose synthesis, we performed molecular docking simulations between TreT 1709.1 and ADP using AlphaFold3 (48). The resulting structural models were visualized using PyMOL v3.1. In addition, conserved domain analysis and visualization were conducted using Batch CD-Search (49) and TBtools-II v2.210 (50).

To verify the functions of stress-resilience genes, RNAi experiments were conducted on five target genes: three TreT paralogs (TreT 1709.1, TreT 6901.1, TreT 9057.1), evm.model.tig639.2 (unannotated), and evm.model.tig2212.1 (protein kinase). To minimize off-target effects, specific DNA fragments (100–120 bp) were designed for each gene and validated by Primer-BLAST against the entire *Apourosomoida* sp. genome to ensure no non-specific matches. These fragments were PCR-amplified with gene-specific primers (Table S23) incorporating restriction SacI and XhoI restriction sites. The amplified products were cloned into the L4440 vector using the ClonExpress^®^ II One Step Cloning Kit (Cat. No. C112; Vazyme). Recombinant plasmids were transformed into *E. coli* HT115 (DE3) competent cells. Transformed bacteria were screened on LB agar plates containing tetracycline (12.5 μg/mL) and ampicillin (50 μg/mL). Positive clones were cultured and induced with 0.4 mM IPTG to express gene-specific dsRNA. To strictly isolate the effect of dsRNA, bacteria carrying the same plasmids but cultured without IPTG induction were used as negative controls. Both IPTG-induced and non-induced bacterial suspensions were washed twice with sterile artificial saline solutions to remove residual antibiotics and IPTG. Ciliates were then fed with either IPTG-induced (RNAi group) or non-induced (control group) *E. coli*. Upon ingestion, *E. coli* cells were lysed in the ciliate cytoplasm, releasing dsRNA to trigger RNAi-mediated degradation of the target mRNA. Ciliates were exposed to each of the three environmental stress conditions—high salinity (80 PSU), hypoxia (0.5 mg/L O₂), or low temperature (4 °C). Each group had three biological replicates. After 24 hours, 100 μL of ciliate culture (including 50 μL of Bouin’s fixative) was collected for microscopic counting of surviving individuals to evaluate the impact of gene knockdown on stress tolerance.

To validate the RNA-seq-based gene expression patterns under hypersaline and hypoxic conditions, and to assess the knockdown efficiency of five target genes following RNAi treatment, we performed RT-qPCR assays. For each replicate of the stress response validation, ~20 lysed cells exposed to 80 PSU or 0.5 mg/L oxygen, along with the untreated control, were collected. For the RNAi efficiency verification, ~20 lysed cells from each replicate of the IPTG-induced (+IPTG) and non-induced (-IPTG) groups were collected for each target gene. The Single Cell Full Length mRNA-Amplification Kit (Cat. No. N711; Vazyme) was used to lyse cells and reverse transcribe the mRNA in the lsysi into cDNA. Three biological replicates were included for each condition. cDNA was also used as the template for qPCR with Hieff UNICON^®^ qPCR SYBR Green Master Mix (Cat. No. 11198ES08; Yeasen) following the manufacturer's protocol. The 18S rRNA gene was used as an internal control. Primers were designed using Primer Premier 6.0 (Premier Biosoft International, Palo Alto, CA) and their specificity was confirmed using Primer-BLAST. Primer sequences are listed in Table S23. Relative gene expression levels were calculated using the 2^-ΔΔCt^ method. For RNAi experiments, the knockdown efficiency was calculated by normalizing the gene expression levels of the induced groups (+IPTG) against the non-induced control groups (-IPTG).

**Measurement of intracellular reactive oxygen species (ROS)**

To evaluate the oxidative stress levels following RNAi-mediated knockdown of the three TreT paralogs (TreT 1709.1, TreT 6901.1, and TreT 9057.1), intracellular ROS production was detected using dihydroethidium (DHE) steining. Cells from both the IPTG-induced RNAi groups (+IPTG) and the non-induced control groups (-IPTG) were harvested by centrifugation. The pellets were fixed with 4% paraformaldehyde (PFA) for 1 h and washed twice with 1× PBS. Subsequently, the cells were resuspended and incubated with 50 μL of DHE working solution (prepared from the Tissue ROS Assay Kit, Cat. No. G1746-100T; Servicebio) and 20 μL of Hoechst 33342 staining solution (Cat. No. C0030; Solarbio). Incubation was performed at 37°C for 30 min in the dark. A negative control group without DHE staining was included to exclude autofluorescence interference.

After washing, cells were mounted and imaged using a Nikon NI-U fluorescence microscope. The red fluorescence (ROS) and blue fluorescence (nuclei) were captured under identical exposure settings (20ms) for all groups. Quantitative analysis of fluorescence intensity was performed using a custom Python script (Github). Briefly, the script performed morphological segmentation to estimate the cell area and calculated the mean red fluorescence intensity per unit area. Experiments were performed in three biological replicates. Statistical significance was determined using a two-tailed Student's t-test, and data are presented as mean ± standard deviation.

**Supplementary Materials References**

1. Z. Lv, L. Chen, L. Y. Chen *et al.*, Morphogenesis and molecular phylogeny of a new freshwater ciliate, *Notohymena apoaustralis* n. sp. (Ciliophora, Oxytrichidae). *J. Eukaryot. Microbiol.* **60**, 455–466 (2013).

2. N. Wilbert, An improved technique of protargol impregnation for ciliates. *Mikrokosmos* **64**, 171–179 (1975).

3. R. Li, W. Zhuang, X. Feng *et al.*, Molecular phylogeny and taxonomy of three anaerobic plagiopyleans (Alveolata: Ciliophora), retrieved from two geographically distant localities in Asia and North America. *Zool. j. Linn. Soc.* **199**, 493–510 (2023).

4. T. Fenchel, B. J. Finlay, Oxygen toxicity, respiration and behavioural responses to oxygen in free-living anaerobic ciliates. *Microbiol.* **136**, 1953–1959 (1990).

5. J. De Gelder, K. De Gussem, P. Vandenabeele *et al.*, Reference database of Raman spectra of biological molecules. *J. Raman Spectrosc.* **38**, 1133–1147 (2007).

6. A. Avetisyan, J. B. Jensen, T. Huser, Monitoring trehalose uptake and conversion by single bacteria using laser tweezers Raman spectroscopy. *Anal. Chem.* **85**, 7264–7270 (2013).

7. X. Sun, X. Zhou, R. Yu *et al.*, Assessing the physiological properties of baker's yeast based on single-cell Raman spectrum technology. *Synth. Syst. Biotechnol.* **10**, 110–118 (2025).

8. H. Li, K. Wu, C. Ruan *et al.*, Cost-reduction strategies in massive genomics experiments. *Mar. Life Sci. Tech.* **1**, 15–21 (2019).

9. S. F. Chen, Y. Q. Zhou, Y. R. Chen *et al.*, fastp: an ultra-fast all-in-one FASTQ preprocessor. *Bioinformatics* **34**, 884–890 (2018).

10. W. De Coster, S. D'Hert, D. T. Schultz *et al.*, NanoPack: visualizing and processing long-read sequencing data. *Bioinformatics* **34**, 2666–2669 (2018).

11. H. Li, R. Durbin, Fast and accurate short read alignment with Burrows-Wheeler transform. *Bioinformatics* **25**, 1754–1760 (2009).

12. H. Li, B. Handsaker, A. Wysoker *et al.*, The sequence alignment/map format and SAMtools. *Bioinformatics* **25**, 2078–2079 (2009).

13. S. Koren, B. P. Walenz, K. Berlin *et al.*, Canu: scalable and accurate long-read assembly via adaptive k-mer weighting and repeat separation. *Genome Res.* **27**, 722–736 (2017).

14. R. Vaser, I. Sovic, N. Nagarajan *et al.*, Fast and accurate *de novo* genome assembly from long uncorrected reads. *Genome Res.* **27**, 737–746 (2017).

15. B. J. Walker, T. Abeel, T. Shea *et al.*, Pilon: an integrated tool for comprehensive microbial variant detection and genome assembly improvement. *PLoS One* **9**, e112963 (2014).

16. C. Camacho, G. Coulouris, V. Avagyan *et al.*, BLAST plus : architecture and applications. *BMC Bioinf.* **10**, 421 (2009).

17. D. F. Guan, S. A. McCarthy, J. Wood *et al.*, Identifying and removing haplotypic duplication in primary genome assemblies. *Bioinformatics* **36**, 2896–2898 (2020).

18. K. Lagesen, P. Hallin, E. A. Rodland *et al.*, RNAmmer: consistent and rapid annotation of ribosomal RNA genes. *Nucleic Acids Res.* **35**, 3100–3108 (2007).

19. M. Manni, M. R. Berkeley, M. Seppey *et al.*, BUSCO update: novel and streamlined workflows along with broader and deeper phylogenetic coverage for scoring of eukaryotic, prokaryotic, and viral genomes. *Mol. Biol. Evol.* **38**, 4647–4654 (2021).

20. A. Mikheenko, A. Prjibelski, V. Saveliev *et al.*, Versatile genome assembly evaluation with QUAST-LG. *Bioinformatics* **34**, 142–150 (2018).

21. N. Chen, Using Repeat Masker to identify repetitive elements in genomic sequences. *Curr. Protoc. Bioinform.* **5**, 4–10 (2004).

22. J. M. Flynn, R. Hubley, C. Goubert *et al.*, RepeatModeler2 for automated genomic discovery of transposable element families. *Proc. Natl. Acad. Sci. USA* **117**, 9451–9457 (2020).

23. P. P. Chan, B. Y. Lin, A. J. Mak *et al.*, tRNAscan-SE 2.0: improved detection and functional classification of transfer RNA genes. *Nucleic Acids Res.* **49**, 9077–9096 (2021).

24. Y. Shulgina, S. R. Eddy, A computational screen for alternative genetic codes in over 250,000 genomes. *eLife* **10**, e71402 (2021).

25. D. Kim, J. M. Paggi, C. Park *et al.*, Graph-based genome alignment and genotyping with HISAT2 and HISAT-genotype. *Nat Biotechnol.* **37**, 907–915 (2019).

26. B. J. Haas, A. Papanicolaou, M. Yassour *et al.*, *De novo* transcript sequence reconstruction from RNA-seq using the Trinity platform for reference generation and analysis. *Nat. Protoc.* **8**, 1494–1512 (2013).

27. T. Bruna, K. J. Hoff, A. Lomsadze *et al.*, BRAKER2: automatic eukaryotic genome annotation with GeneMark-EP plus and AUGUSTUS supported by a protein database. *NAR Genom. Bioinform.* **3**, lqaa108 (2021).

28. E. Sallet, J. Gouzy, T. Schiex, EuGene: an automated integrative gene finder for eukaryotes and prokaryotes. *Methods Mol. Biol.* **1962**, 97–120 (2019).

29. B. J. Haas, S. L. Salzberg, W. Zhu *et al.*, Automated eukaryotic gene structure annotation using EVidenceModeler and the program to assemble spliced alignments. *Genome Biol.* **9**, R7 (2008).

30. S. Gotz, J. M. Garcia-Gomez, J. Terol *et al.*, High-throughput functional annotation and data mining with the Blast2GO suite. *Nucleic Acids Res.* **36**, 3420–3435 (2008).

31. C. P. Cantalapiedra, A. Hernández-Plaza, I. Letunic *et al.*, eggNOG-mapper v2: functional annotation, orthology assignments, and domain prediction at the metagenomic scale. *Mol. Biol. Evol.* **38**, 5825–5829 (2021).

32. M. Tillich, P. Lehwark, T. Pellizzer *et al.*, GeSeq–versatile and accurate annotation of organelle genomes. *Nucleic Acids Res.* **45**, W6–W11 (2017).

33. S. Kovaka, A. V. Zimin, G. M. Pertea *et al.*, Transcriptome assembly from long-read RNA-seq alignments with StringTie2. *Genome Biol.* **20**, 278 (2019).

34. M. I. Love, W. Huber, S. Anders, Moderated estimation of fold change and dispersion for RNA-seq data with DESeq2. *Genome Biol.* **15**, 550 (2014).

35. G. C. Yu, L. G. Wang, Y. Y. Han *et al.*, clusterProfiler: an R package for comparing biological themes among gene clusters. *OMICS* **16**, 284–287 (2012).

36. I. Kerseviciute, J. Gordevicius, aPEAR: an R package for autonomous visualization of pathway enrichment networks. *Bioinformatics* **39**, btad672 (2023).

37. D. M. Emms, S. Kelly, OrthoFinder: phylogenetic orthology inference for comparative genomics. *Genome Biol.* **20**, 238 (2019).

38. Z. Zhang, J. F. Xiao, J. Y. Wu *et al.*, ParaAT: a parallel tool for constructing multiple protein-coding DNA alignments. *Biochem. Bioph. Res. Co.* **419**, 779–781 (2012).

39. B. Q. Minh, H. A. Schmidt, O. Chernomor *et al.*, IQ-TREE 2: new models and efficient methods for phylogenetic inference in the genomic era. *Mol. Biol. Evol.* **37**, 2461–2461 (2020).

40. Z. H. Yang, PAML 4: phylogenetic analysis by maximum likelihood. *Mol. Biol. Evol.* **24**, 1586–1591 (2007).

41. F. K. Mendes, D. Vanderpool, B. Fulton *et al.*, CAFE 5 models variation in evolutionary rates among gene families. *Bioinformatics* **36**, 5516–5518 (2020).

42. Z. H. Yang, R. Nielsen, Estimating synonymous and nonsynonymous substitution rates under realistic evolutionary models. *Mol. Biol. Evol.* **17**, 32–43 (2000).

43. M. Lynch, B. Walsh, *Genetics and analysis of quantitative traits* (Sinauer, Sunderland, Massachusetts, 1998), vol. 1, pp. 818.

44. X. X. Shen, D. A. Opulente, J. Kominek *et al.*, Tempo and mode of genome evolution in the budding yeast subphylum. *Cell* **175**, 1533–1545 (2018).

45. G. Schönknecht, W. H. Chen, C. M. Ternes *et al.*, Gene transfer from bacteria and archaea facilitated evolution of an extremophilic eukaryote. *Science* **339**, 1207–1210 (2013).

46. F. D. K. Tria, G. Landan, T. Dagan, Phylogenetic rooting using minimal ancestor deviation. *Nat Ecol Evol* **1**, 0193 (2017).

47. D. Bryant, M. Charleston, MAD roots for large trees. *arXiv*, 1811.03174 (2018).

48. J. Abramson, J. Adler, J. Dunger *et al.*, Accurate structure prediction of biomolecular interactions with AlphaFold 3. *Nature* **630**, 493–500 (2024).

49. A. Marchler-Bauer, S. Lu, J. B. Anderson *et al.*, CDD: a conserved domain database for the functional annotation of proteins. *Nucleic Acids Res.* **39**, D225–D229 (2010).

50. C. Chen, Y. Wu, J. Li *et al.*, TBtools-II: A “one for all, all for one” bioinformatics platform for biological big-data mining. *Mol. Plant* **16**, 1733–1742 (2023).

**
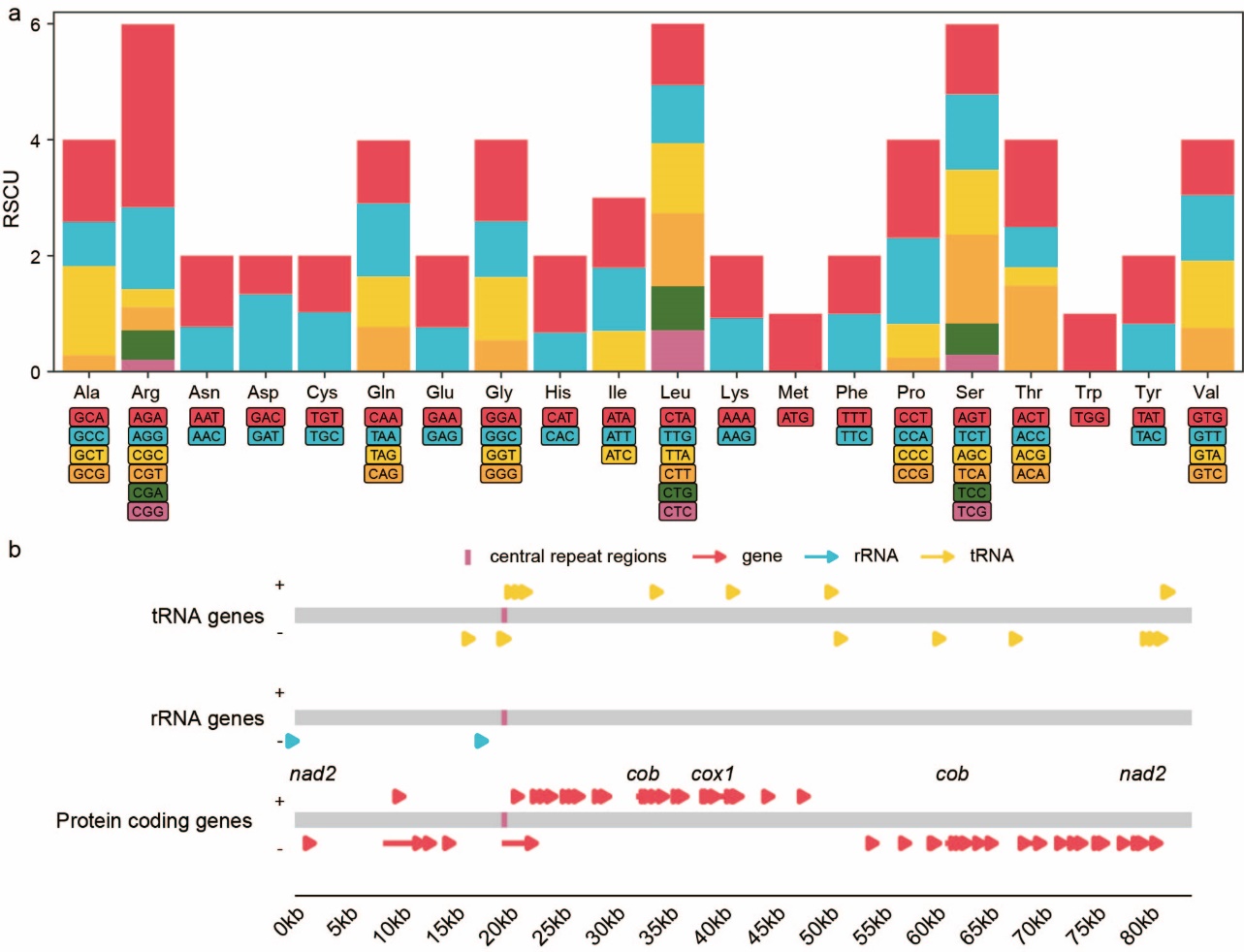
Supplementary Figures**

Fig. S1. Sampling site and genomic features of *Apourosomoida* sp. (a) Relative synonymous codon usage (RSCU) distribution. (b) Annotated mitochondrial genome structure, including protein coding genes (red), transfer RNAs (yellow), and ribosomal RNAs (blue).

**
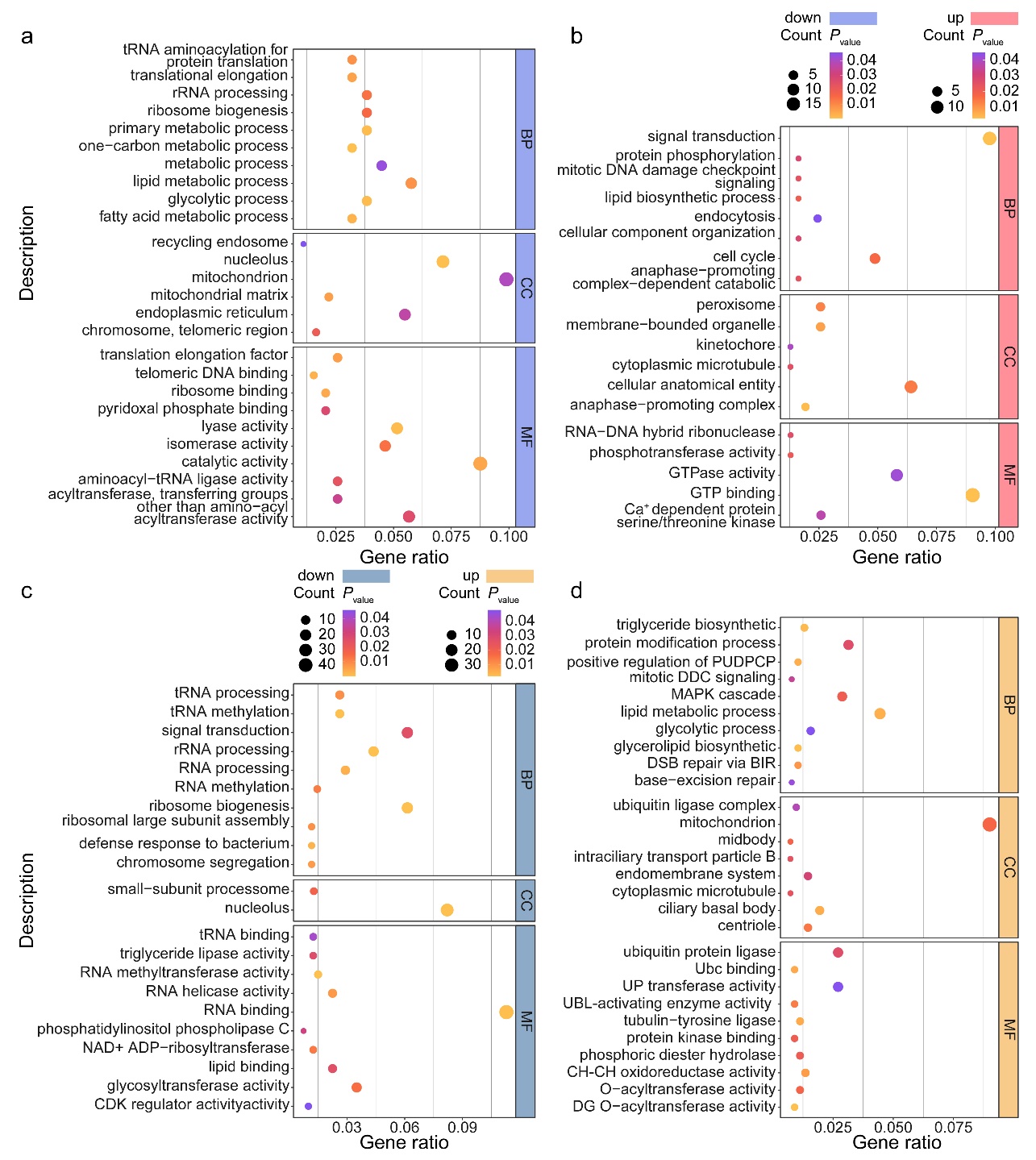
**

Fig. S2. Pathway enrichment of DEGs under stress treatments. (a) GO enrichment of significantly down-regulated and (b) up-regulated DEGs under low-temperature treatment (4 °C). (c–d) GO enrichment of significantly down-regulated (c) and up-regulated (d) DEGs under hypoxic treatment (0.5 mg/L).


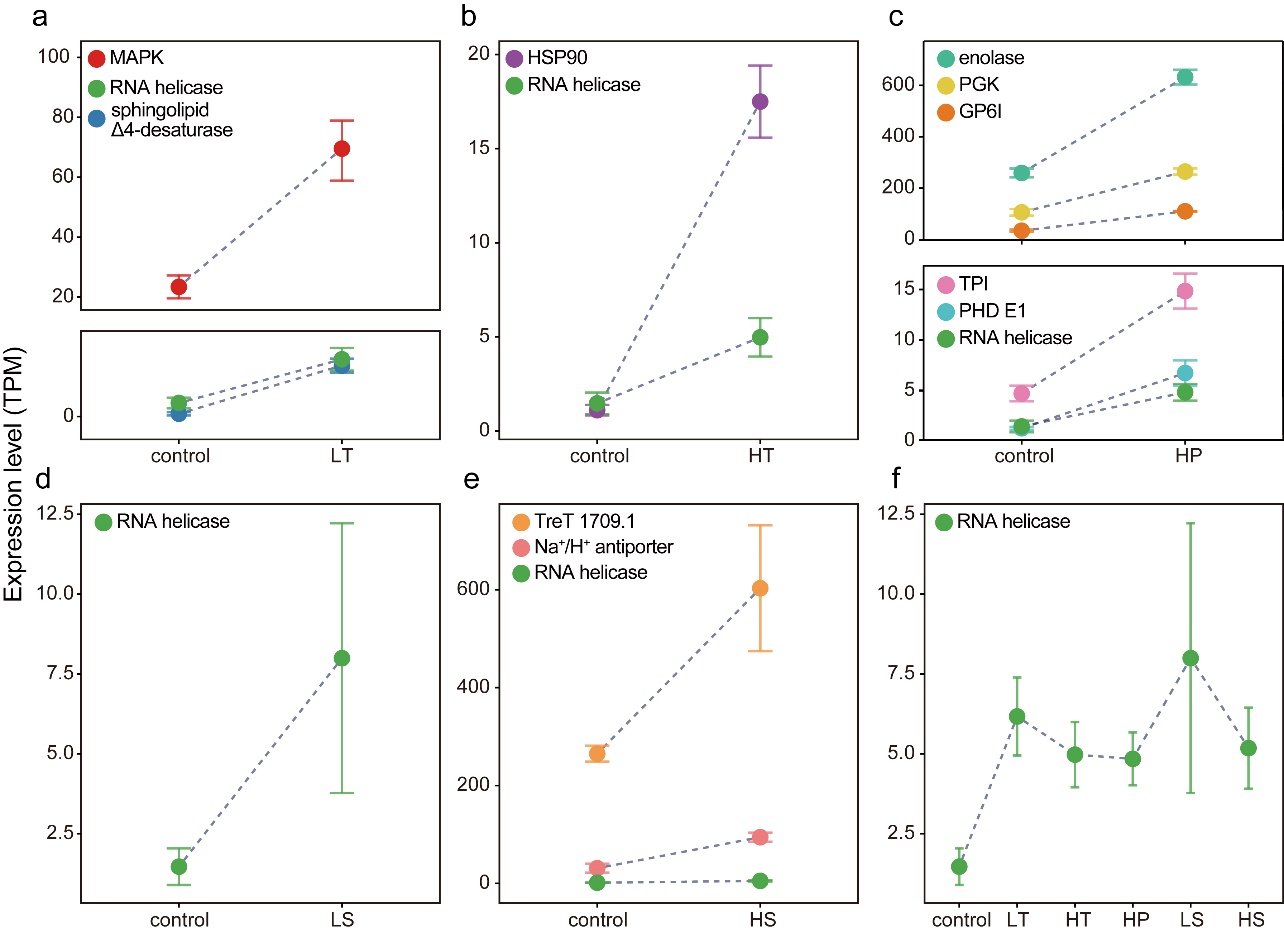


Fig. S3. Stress-induced transcriptional profiling of *Apourosomoida* sp. (a) Significantly up-regulated DEGs under low temperature (4 °C): MAPK protein kinase (evm.model.tig5006.1) and sphingolipid Δ4-desaturase (evm.model.tig3865.1). (b) Significantly up-regulated HSP90 (evm.model.tig8570.3) under high temperature (28 °C). (c) Up-regulated genes under hypoxia: GPI (evm.model.tig8578.1), PGK (evm.model.tig4859.1), enolase (evm.model.tig2054.1), TPI (evm.model.tig5463.1), and PHD E1 (evm.model.tig8149.1). (d) RNA helicase (evm.model.tig9617.1) under low salinity. (e) Up-regulated TreT (evm.model.tig1709.1) and Na⁺/H⁺ antiporter (evm.model.tig305.2) under high salinity (80 PSU). (f) Expression levels of RNA helicase (evm.model.tig9617.1) across treatments: LT-low temperature (4 °C), HT-high temperature (28 °C), HP-hypoxia (0.5 mg/L), LS-low salinity (20 PSU), HS-high salinity (80 PSU) and the control. All expression levels are in TPM (Transcripts Per Million).


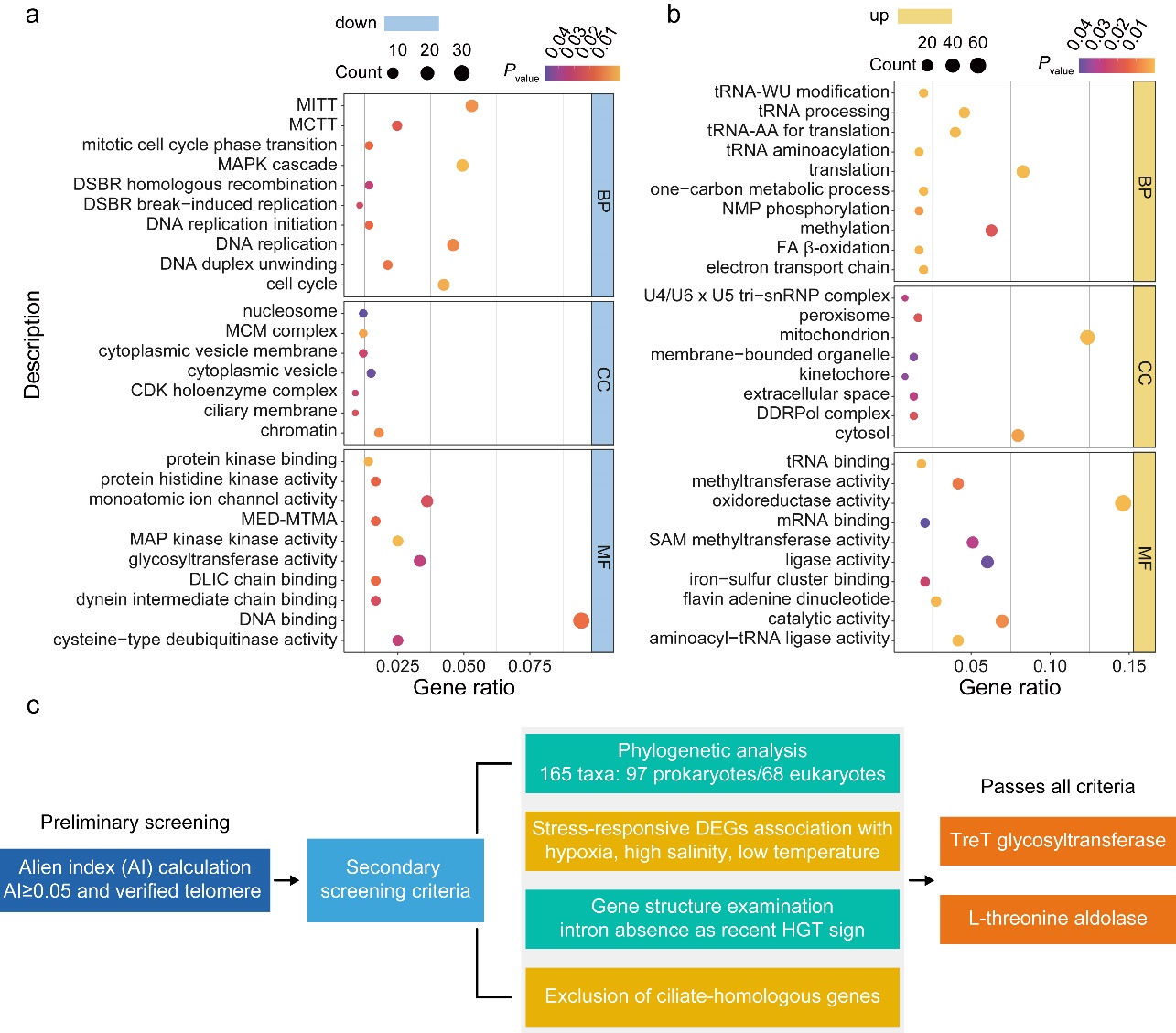


Fig. S4. GO enrichment analysis of DEGs under high-salinity treatment (80 PSU) and HGT analysis workflow. (a) Results based on significantly down-regulated DEGs. (b) Results based on significantly up-regulated DEGs. (c) Schematic workflow for identifying HGT genes in *Apourosomoida* sp. 
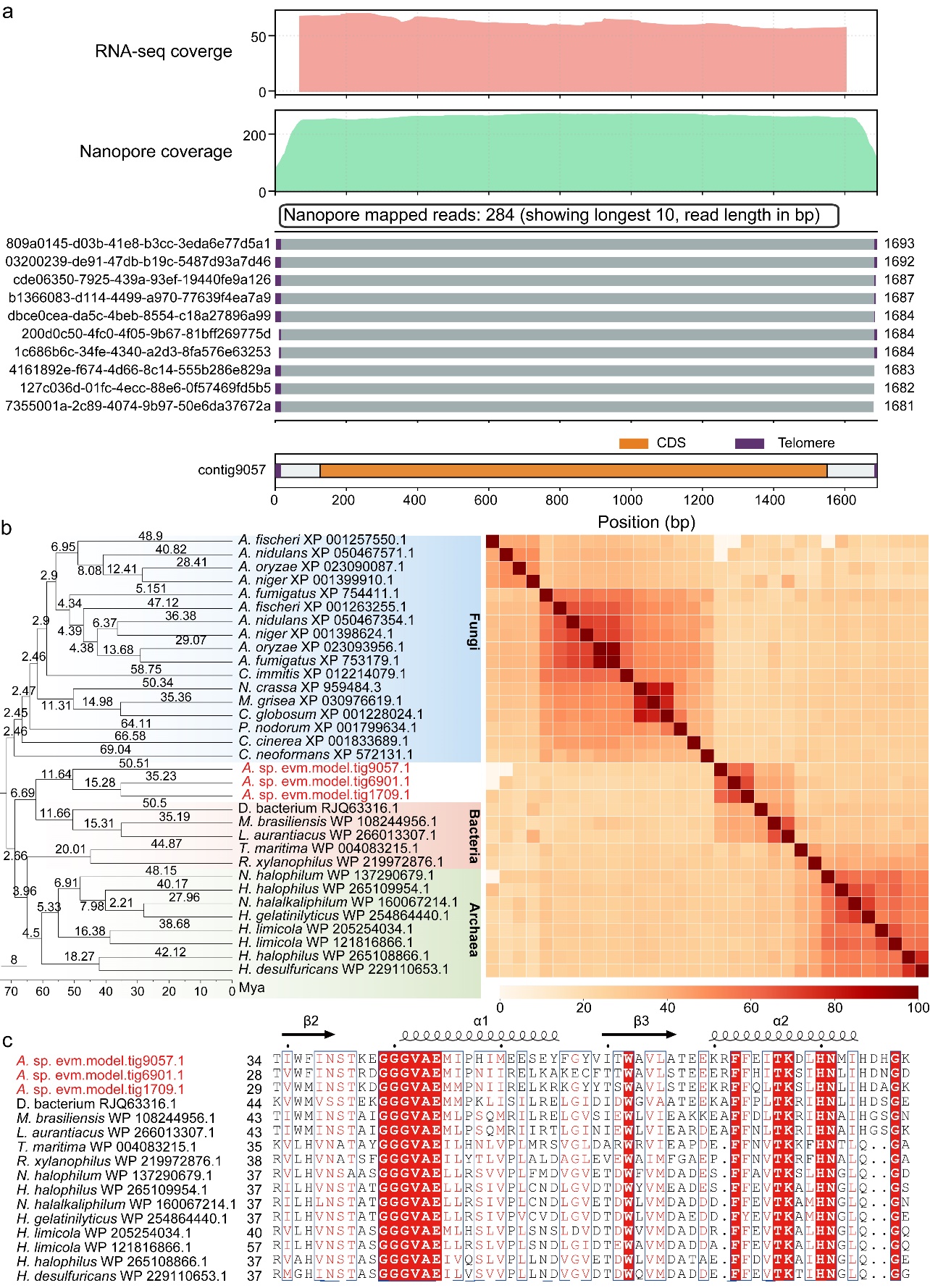


Fig. S5. Divergence and structural analyses of the TreT gene family. (a) Information for validating HGT. Top: Uniform coverage distributions of Illumina RNA-seq (pink) and Nanopore DNA-seq (green). Middle: Alignment of raw Nanopore reads spanning the full chromosome length, including some with telomeric ends. Bottom: Schematic of contig9057 (1,695 bp) showing the TreT CDS (orange) flanked by telomeric repeats (C_4_A_4_)_n_, (purple). Mean sequencing quality scores of the top 10 longest Nanopore reads: 16.40 (SD=2.11) or 97.43% accuracy. (b) Divergence time estimation and pairwise sequence identity of TreT homologs. In the time-calibrated tree (left), *Aspergillus niger* vs. *Aspergillus fumigatus* was used as the calibration point. The axis at the bottom represents time in million years ago (Mya), and numbers on branches indicate node-specific divergence times. The heatmap on the right displays the pairwise amino acid identity values between sequences. Identity scores were calculated using Blastp with an e-value cutoff of 10. Blank or missing values indicate alignments with e-value > 10 (no significant similarity). (c) Amino acid alignment of TreT glycosyltransferase genes in *Apourosomoida* sp. (33–63 amino acid region) with bacterial and archaeal homologs, with secondary structure annotations (α-helices, β-sheets) above the sequence alignment. Blue boxes indicate regions where sequence identity between groups exceeds 70%.


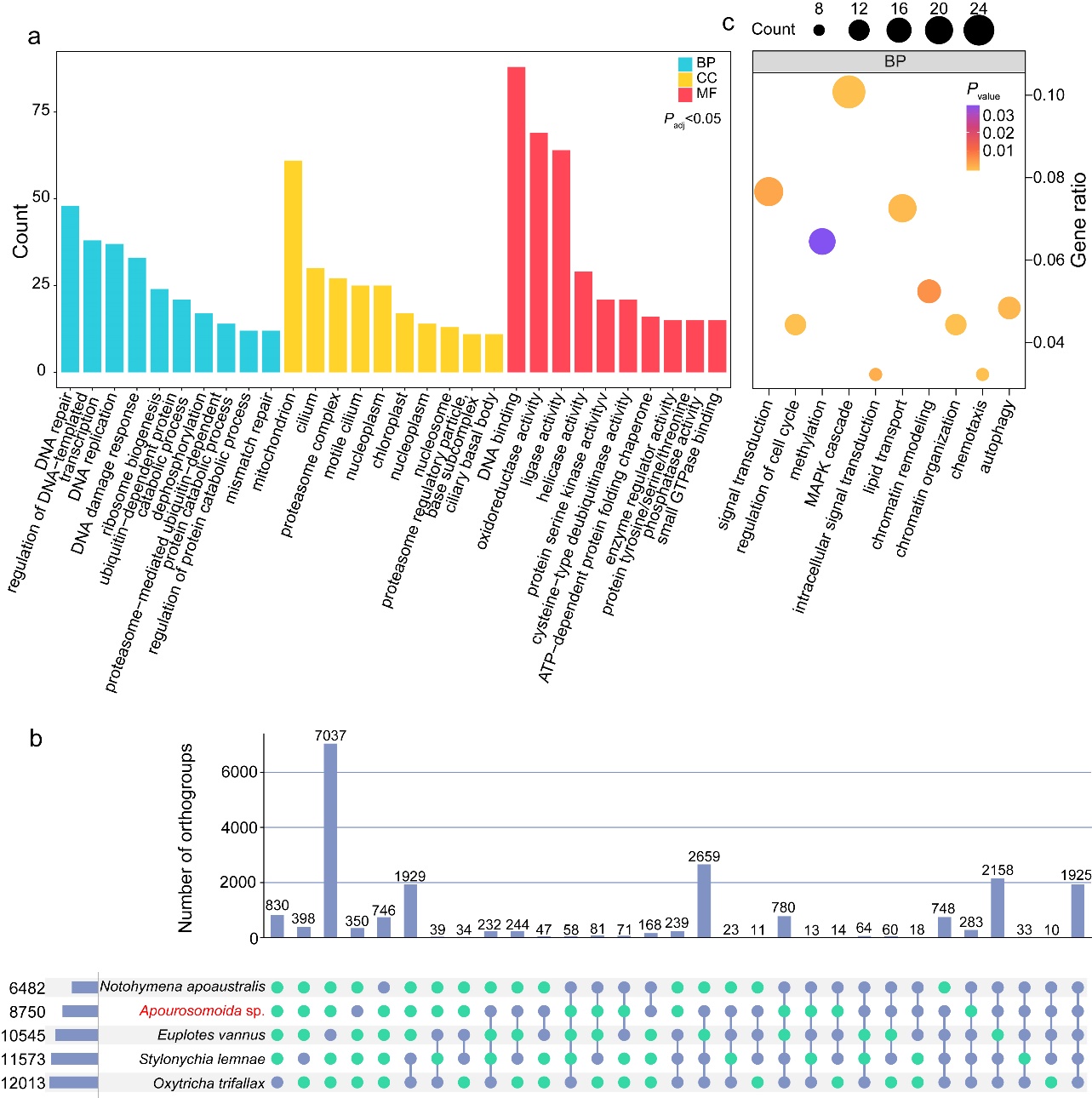


Fig. S6. Comparative genomics and functional enrichment analysis. (a) GO enrichment analysis of the top 10 expanded gene families in *Apourosomoida* sp. (b) Homologous gene family distribution across five Spirotrichea species. (c) GO enrichment analysis of the top 10 unique gene families in *Apourosomoida* sp.


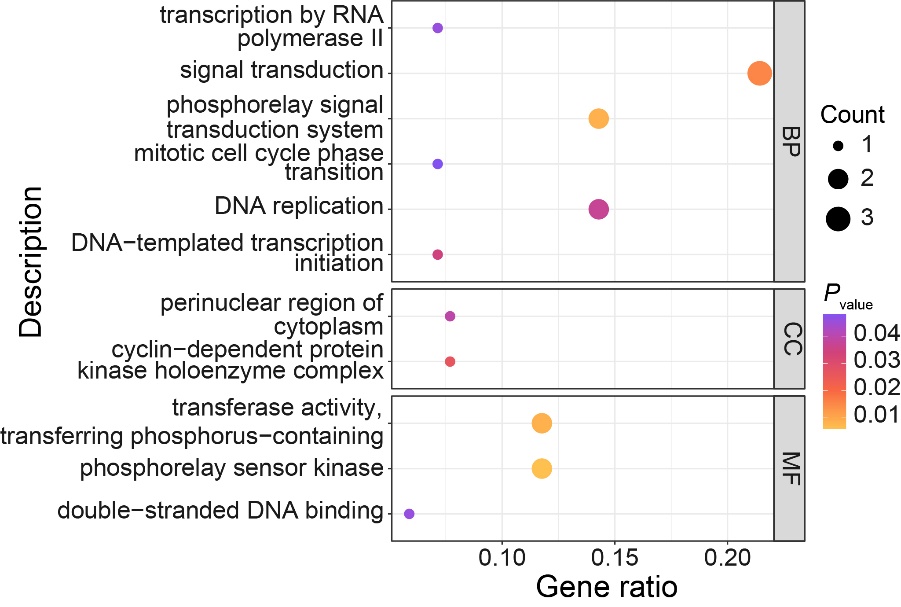


Fig. S7. GO enrichment analysis of the top 20 dN-ranked genes also differentially expressed under environmental stress in *Apourosomoida* sp.


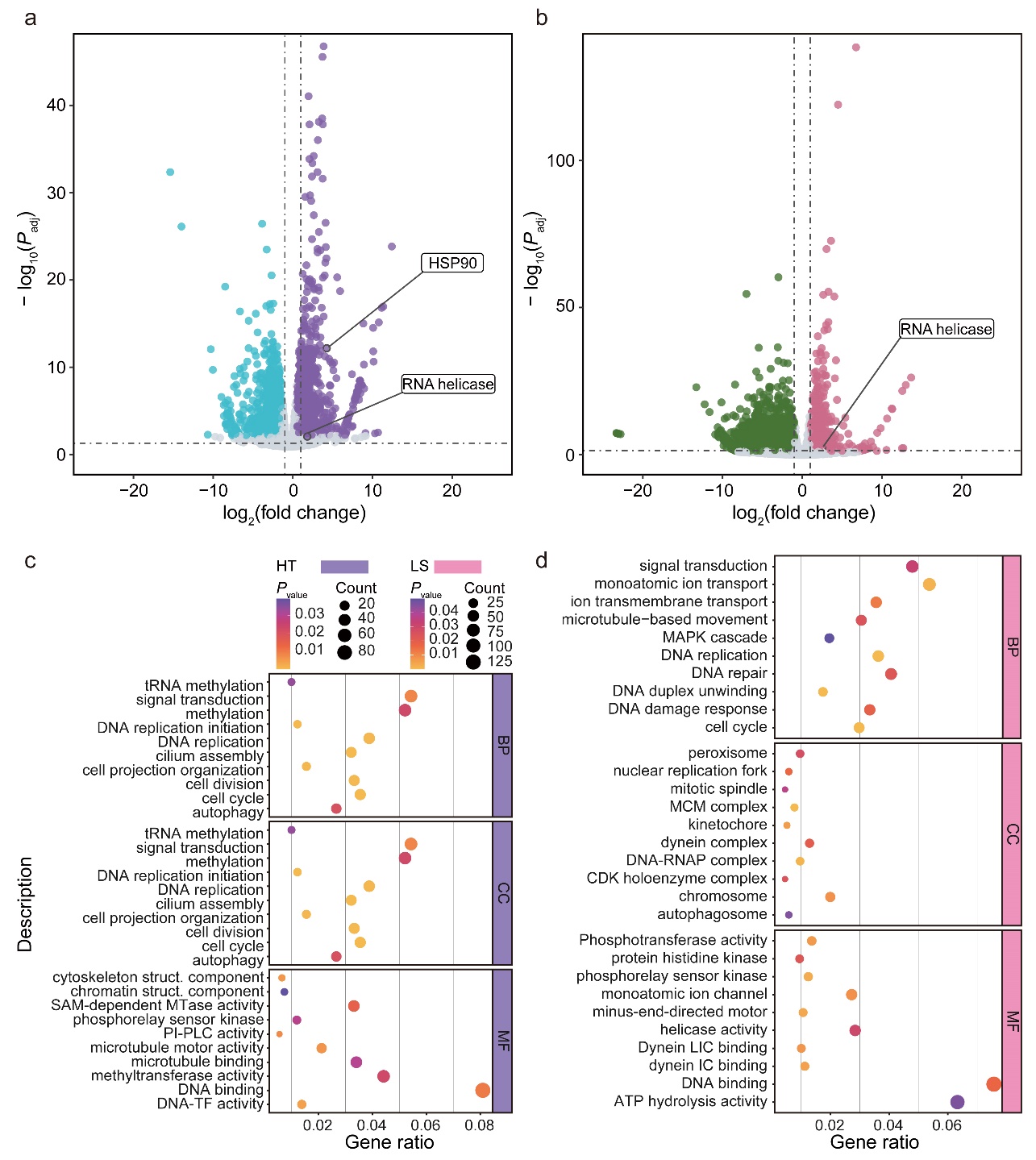


Fig. S8. Transcriptomic analysis under thermal or hyposaline stress in *Apourosomoida* sp. (a) Volcano plot of DEGs under high-temperature treatment (28 °C). (b) Volcano plot of DEGs under low-salinity treatment (20 PSU). (c) GO enrichment of significant DEGs under high-temperature treatment. (d) GO enrichment of significant DEGs under low-salinity treatment.

**
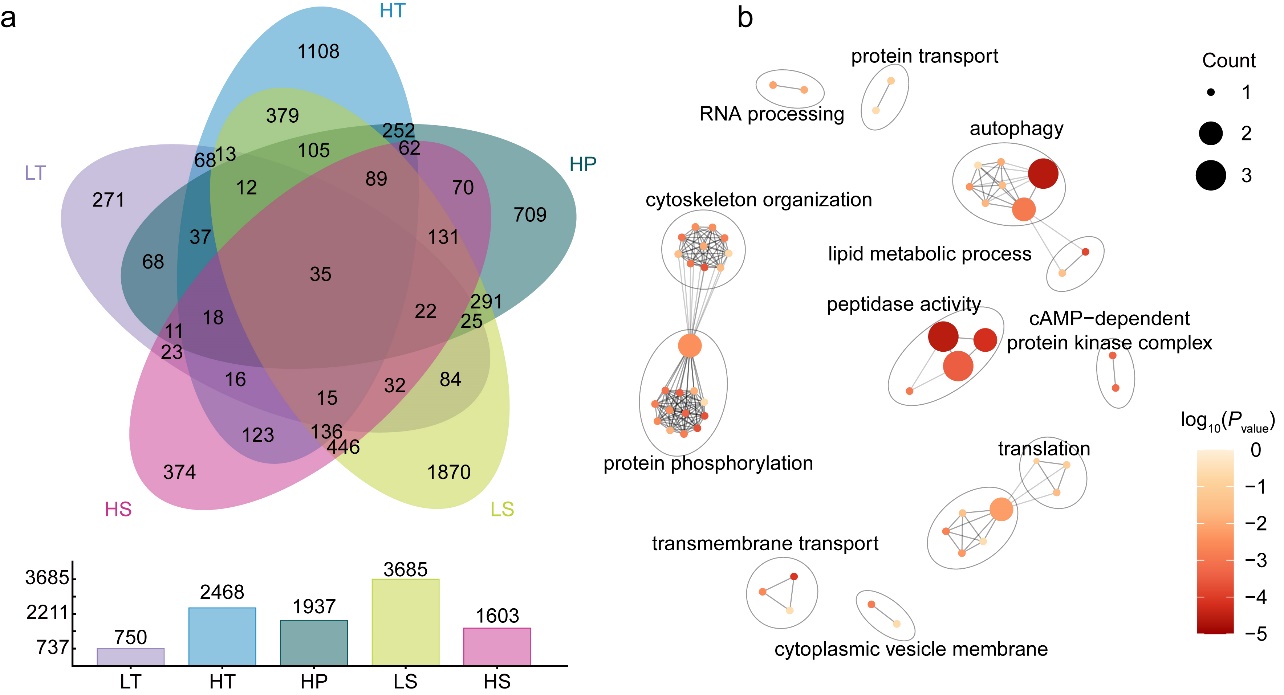
**

Fig. S9. Stress-responsive genes and functional networks in *Apourosomoida* sp. (a) Venn diagram showing 35 shared DEGs up-regulated/down-regulated across five stress treatments: low salinity (LS, 20 PSU), high salinity (HS, 80 PSU), hypoxia (HP, 0.5 mg/L), low temperature (LT, 4 °C), and high temperature (HT, 28 °C). (b) Pathway enrichment network illustrates biological functions of the 35 shared DEGs, dot size corresponds to gene number in the pathway, color intensity refers to log_10_(*P*_value_) from GO analyses. Clusters were generated from enriched GO terms by aPEAR package.
